# Plant negative-strand RNA virus phosphoprotein condensates exploit host trafficking and lipid synthesis for viral factory assembly

**DOI:** 10.1126/sciadv.adx7905

**Published:** 2025-08-20

**Authors:** Zhiyi Wang, Jingyi Zhang, Jilei Huang, Gan Sha, Xinyue Song, Xue Cao, Zhenchen Yan, Chuanhe Liu, Siping Chen, Ziying Li, Xiuqin Huang, Qingjun Xie, Xin Yang, Guohui Zhou, Tong Zhang

**Affiliations:** ^1^ Guangdong Basic Research Center of Excellence for Precise Breeding of Future Crops, South China Agricultural University, Guangzhou 510642, China.; ^2^ Guangdong Province Key Laboratory of Microbial Signals and Disease Control, College of Plant Protection, South China Agricultural University, Guangzhou 510642, China.; ^3^ National Key Laboratory of Green Pesticide, South China Agricultural University, Guangzhou 510642, China.; ^4^ Instrumental Analysis and Research Center, South China Agricultural University, Guangzhou 510642, China.; ^5^ Guangdong Provincial Key Laboratory of Plant Molecular Breeding, College of Agriculture, South China Agricultural University, Guangzhou 510642, China.

## Abstract

RNA viruses often remodel host intracellular membranes to establish specialized replication compartments through viral protein–induced phase separation. However, the mechanisms underlying membrane remodeling and the characteristics that render these sites conducive to replication remain poorly understood, particularly in plant negative-strand RNA viruses. Here, we demonstrate that the phosphoprotein (P) of rice stripe mosaic virus (RSMV) forms biomolecular condensates via liquid-liquid phase separation (LLPS) to recruit essential components for viral replication factories (VFs). We identify a direct interaction between RSMV P and adenosine diphosphate (ADP) ribosylation factor 1 (OsARF1C), a crucial regulator of the coatomer protein I (COP I) vesicle transport pathway that is vital for viral replication. This interaction indirectly recruits OsARF1C’s partner, phosphatidylinositol 4-kinase beta (OsPI4KB), which drives localized phosphatidylinositol-4 phosphate (PI4P) synthesis. Concurrently, the P protein modulates its aggregates and LLPS droplets through PI4P, thereby expanding the replication site and enhancing viral replication.

## Teaser

RSMV P forms phase-separated condensates recruiting COP I to synthesize PI4P, expanding viral replication sites for infection.

## INTRODUCTION

Biomolecular condensates, driven by liquid-liquid phase separation (LLPS), are diverse membraneless organelles within cells that can move freely without being confined by a double-layer membrane. These structures play crucial roles in various cellular processes in eukaryotic cells, including growth, development, and signal transduction (*1*–*3*). Identified biomolecular condensates in cells include the nucleolus, Cajal bodies, processing bodies, stress granules, and more (*4*, *5*). Research indicates that the formation of LLPS is primarily regulated by intrinsically disordered regions (IDRs) of proteins. Furthermore, LLPS is influenced by posttranslational modifications of proteins, host factors, and changes in cellular environments (*2*, *3*, *6*, *7*).

During the infection of host cells, many viruses create unique organelles that consist of viral proteins, host factors, and nucleic acids. These organelles, commonly referred to as viral inclusion bodies, viroplasms, or viral factories (VFs), play crucial roles in viral replication, transcription, and assembly (*8*). The formation of VFs exhibits a high level of dynamic adaptability, rapidly assembling and disassembling throughout the viral infection process. Materials continuously flow and exchange within and outside these inclusion bodies, suggesting a close resemblance to biomolecular condensates driven by LLPS (*9*). A growing body of evidence shows that viruses use their encoded proteins to undergo LLPS, establishing liquid condensates that serve as VFs. For instance, positive-sense RNA viruses, such as severe acute respiratory syndrome coronavirus 2 (SARS-CoV-2), which encodes the nucleocapsid (N) protein (*10*–*12*), as well as human norovirus (HuNoV) that encodes RNA-dependent RNA polymerase (RdRp) (*13*), are known to form such structures. Negative-sense RNA viruses, including Ebola virus (*14*), vesicular stomatitis virus (*15*), respiratory syncytial virus (RSV) (*16*), and rabies virus (RABV) (*17*, *18*), also participate in LLPS through proteins such as N protein and phosphoprotein (P), while double-stranded RNA viruses such as rotavirus, which encodes the nonstructural protein NSP5, are capable of forming viroplasms essential for viral replication (*19*). Similar mechanisms have recently been corroborated in plant viruses, where inhibiting the phase separation of the tomato spotted wilt virus N protein effectively blocked viral infection (*20*). Notable progress has been made in understanding LLPS of plant viral proteins, suggesting that viral condensates may enhance virus fitness by facilitating biomolecule concentration and improving the efficiency of energy-intensive processes such as RNA replication (*21*). The barley yellow striate mosaic virus (BYSMV) P protein dynamically coordinates viral replication and transcription through phosphorylation-modulated phase separation dynamics (*22*). The tomato yellow mottle-associated virus uses its N and P proteins to assemble viral replication factories exhibiting LLPS properties (*23*). The tomato bushy stunt virus (TBSV) p33 and the carnation Italian ringspot virus p36 facilitate viral replication factory assembly via LLPS (*24*). These findings highlight the dual role of viral protein–driven LLPS in assembling inclusion bodies and expediting viral replication. However, the regulatory factors and precise mechanisms governing LLPS-mediated assembly of viral replication factories remain key unresolved questions in the field.

RNA viruses often modify intracellular membranes to create replication sites characterized by unique lipids and proteins distinct from those of the original organelles (*25*, *26*). Phosphoinositides (PIPs) are recognized lipids involved in the replication of various RNA viruses (*27*–*29*). They are produced through the phosphorylation of phosphatidylinositol (PI) at the D-3, D-4, and/or D-5 positions of the hydroxyl groups in its inositol ring (*30*–*32*). Phosphatidylinositol 4-phosphate (PI4P) is the predominant PIP in cells, widely distributed across various cellular membranes, with the highest abundance at the trans-Golgi network (TGN) (*33*). PI4P is synthesized by phosphatidylinositol 4-kinases (PI4Ks). Mammals possess four PI4K isoforms, namely, the III isoforms PI4KA and PI4KB, and the II isoforms PI4K2A and PI4K2B (*32*, *34*, *35*). In plants, only the III isoforms PI4KA/B have been confirmed to exhibit kinase activity in generating PI4P (*36*). PI4KA primarily localizes to the endoplasmic reticulum (ER), regulating the exit of transport vesicles from the ER, while PI4KB governs transport at the Golgi apparatus and from the Golgi to the plasma membrane (*37*). Studies have shown that many animal RNA viruses can directly recruit PI4KB, relying on its activity to generate abundant PI4P to facilitate viral replication. In addition, plant positive-sense RNA viruses, such as TBSV, can enhance their own replication by hijacking different vesicular transport pathways or directly recruiting PI4K to produce ample PI4P (*27*, *29*, *38*–*42*). Furthermore, several studies indicate that viruses can indirectly recruit the PI4KB-PI4P pathway by enlisting other host factors. Among these, research suggests that adenosine diphosphate (ADP) ribosylation factor 1 (ARF1), a key factor in coatomer protein I (COP I) vesicle formation, can recruit PI4KB to the Golgi apparatus, stimulating PI4KB to generate PI4P for the budding of Golgi-derived transport vesicles (*27*, *42*, *43*). To date, it remains unclear whether PIPs are necessary for the replication of plant negative-sense RNA viruses and how they are recruited or synthesized at replication sites.

Rice stripe mosaic virus (RSMV) is a notable pathogen responsible for considerable losses in rice production (*44*). It belongs to the family Rhabdoviridae, genus *Cytorhabdovirus*, and has a genome comprising a negative-sense single-stranded RNA of ~13 kb (*45*). The complementary strand of the genome encodes seven nonoverlapping open reading frames that produce the classic N, P, matrixprotein (M), glycoprotein (G), and largeprotein (L) proteins of rhabdoviruses, along with two accessory proteins (*45*–*47*). Our recent study successfully established a mini-replicon (MR) system for RSMV that replicates and transcribes within *Nicotiana benthamiana* cells. Through this system, we found that the P protein encoded by RSMV can form granules, which are crucial for viral replication (*48*). Understanding the composition of the RSMV VFs and analyzing the factors that influence their dynamic adaptability may help in developing a theoretical model for the replication of plant negative-sense RNA viruses as well as provide new targets for disease prevention and control. In this study, we demonstrated that the P protein encoded by RSMV forms the core of the VFs through LLPS. Moreover, we identified that the P protein indirectly recruits OsPI4KB into the VFs by interacting with OsARF1C, a key factor in the vesicle transport pathway. This interaction facilitates the generation and accumulation of PI4P within the VFs. The presence of PI4P enhances the formation of larger granules during P protein LLPS, thereby providing RSMV with expanded replication sites and accelerating the viral infection process.

## RESULTS

### The P protein is a crucial component of the VFs formed during RSMV infection

**Fig. 1.**
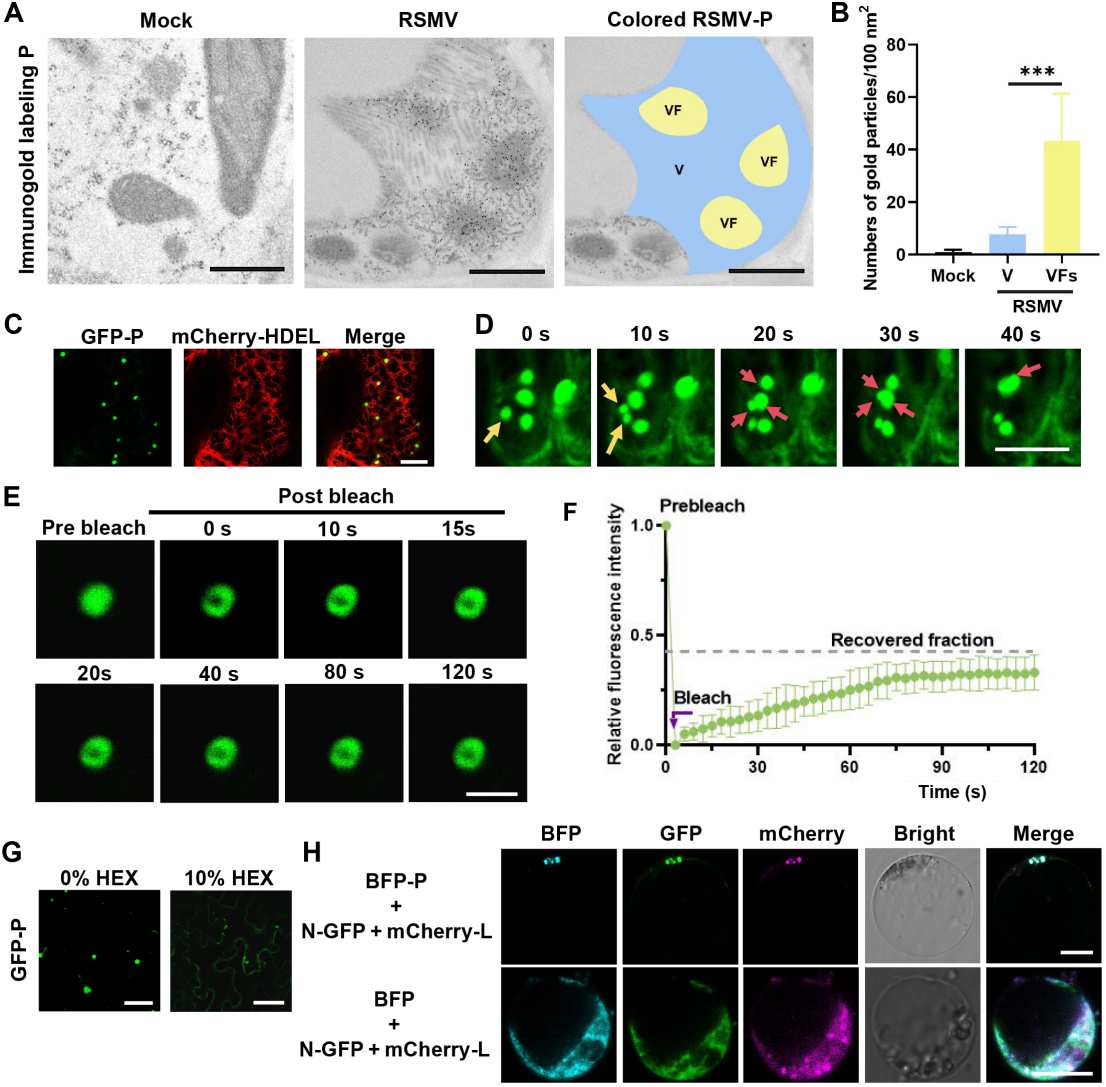
RSMV P protein enriches in VFs and undergoes LLPS in vivo. (**A**) Immunosorbent electron microscopy analysis of P subcellular localization patterns in noninfected and RSMV-infected rice stem cells at 30 days post inoculation (dpi). P is labeled with 12-nm gold particles. The blue-labeled areas represent virus particles (V), while the yellow-labeled areas represent VFs. Scale bars, 200 nm. (**B**) The number of P protein colloidal gold particles in both V and VF regions. Gold particles were counted in 30 cells; statistical analysis was conducted using Student’s *t* test, with *** indicating *P* < 0.001. (**C**) Localization of GFP-P and mCherry-HDEL expressed in *N. benthamiana* leaves at 55 hours post infiltration (hpi). Scale bar, 20 μm. (**D**) Confocal images showing the fusion of two GFP-P granules in *N. benthamiana* leaf epidermal cells, with red arrows indicating granule fusion and yellow arrows indicating granule split. Scale bar, 2 μm. (**E**) Confocal images illustrating FRAP of GFP-P granules in *N. benthamiana* leaves at 55 hpi. Scale bar, 5 μm. (**F**) FRAP recovery curves for GFP-P granules, with the intensity of each granule normalized to its prebleach fluorescence. Data are presented as means ± SD of 10 granules. (**G**) Representative confocal images of GFP-P droplets before and after treatment with 10% HEX for 10 min in *N. benthamiana* leaves. Scale bars, 20 μm. (**H**) Subcellular distribution of BFP-P, N-GFP, and mCherry-L in rice protoplasts. Scale bars, 10 μm.

In our previous study, we identified that the RSMV-encoded P protein forms VF-like inclusion bodies that are critical for viral replication (*48*). Electron microscopy revealed numerous ring-shaped VFs in the cytoplasm, encircling newly synthesized viral ribonucleoproteins (RNPs) (fig. S1A). Immunolabeling with a gold-conjugated P-specific antibody demonstrated a concentrated distribution of gold particles within the VFs, in contrast to their sparse presence in other virus-infected regions (Fig. 1A). Quantitative analysis of gold-labeled P protein within designated regions further substantiated these observations (Fig. 1B), highlighting the notable role of the P protein in RSMV VF architecture.

To characterize the material properties of the RSMV P, we examined the subcellular localization of green fluorescent protein (GFP)–P in *N. benthamiana* leaves. Consistent with previous findings (*48*), GFP-P formed granules on the ER (Fig. 1C). These granules exhibited dynamic movement within the cytoplasm, undergoing fusion and division (Fig. 1D and movie S1). Fluorescence recovery after photobleaching (FRAP) revealed that ~36% of the GFP-P granule signal recovered within 120 s, indicating rapid protein redistribution between membraneless granules and cellular proteins (Fig. 1, E and F). Treatment with 1,6-hexanediol (HEX), a chemical inhibitor of liquid-like droplets, effectively dispersed the GFP-P granules (Fig. 1G). These findings suggest that the RSMV P protein forms liquid-like granules in vivo.

During rhabdovirus infection, viroplasms primarily comprise N, P, and L proteins for viral replication and transcription, and P functions as a central protein that anchors the polymerase complexes to the N-RNA templates (*49*). Our investigation focused on the potential role of RSMV P in facilitating the recruitment of N and L proteins for efficient RNA synthesis. Coexpression experiments in rice protoplasts revealed that N-GFP was incorporated into blue fluorescent protein (BFP)-P granules but not free BFP (fig. S1B). Similarly, BFP-P bodies were found to recruit mCherry-L, rather than mCherry (fig. S1C). Coexpression of N-GFP, mCherry-L, and BFP-P in rice protoplasts resulted in the formation of spherical granules, while coexpression with BFP led to even distribution in the cytoplasm (Fig. 1H). These results indicate that the RSMV P protein acts as a scaffold protein, initiating granule formation and facilitating the recruitment of N protein into condensed RNP complexes.

### RSMV P undergoes phase separation in vitro

**Fig. 2.**
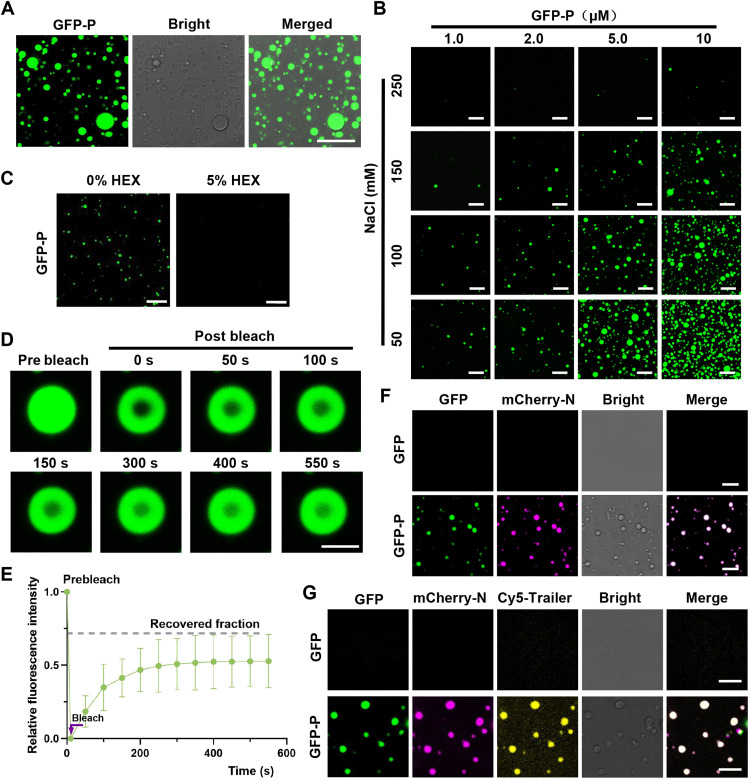
P protein undergoes LLPS and recruits the N protein and genomic RNA in vitro. (**A**) Confocal images showing droplets formed by GFP-P at a concentration of 10 μM in 100 mM NaCl. Scale bar, 10 μm. (**B**) Confocal images demonstrating phase separation of GFP-P at various concentrations of GFP-P and NaCl. Scale bars, 20 μm. (**C**) Confocal images illustrating GFP-P droplets before and after treatment with 5% HEX for 1 min. Scale bars, 20 μm. (**D**) Confocal images displaying FRAP of GFP-P droplets in vitro at a concentration of 10 μM in 100 mM NaCl. Scale bar, 2 μm. (**E**) FRAP recovery curve of GFP-P droplets, with data represented as the means ± SD of 10 droplets. (**F**) Incorporation of mCherry-N into GFP-P droplets, where free GFP could not form droplets to recruit mCherry-N. Scale bars, 20 μm. (**G**) Confocal images representing the incorporation of mCherry-N and Cy5-Trailer of the RSMV genome into GFP-P droplets, where free GFP was unable to form droplets to recruit mCherry-N and Cy5-Trailer. Scale bars, 20 μm.

The spherical GFP-P foci exhibited characteristics indicative of typical phase-separated biomolecular condensates. In vitro experiments confirmed the phase separation of the purified recombinant GFP-P protein, resulting in the formation of spherical droplets (Fig. 2A). Both the number and size of droplets increased with higher GFP-P concentrations and lower NaCl concentrations (Fig. 2B). HEX treatment effectively dispersed the GFP-P droplets (Fig. 2C). FRAP analysis revealed a recovery of ~50% of GFP-P fluorescent signals within 550 s (Fig. 2, D and E). Low-complexity regions or IDRs are well-established drivers of phase separation (*7*). Previous findings identified three IDRs in the RSMV P protein, with IDR2 playing a crucial role in the formation of GFP-P droplets (*43*). The deletion of IDR2 from the recombinant protein (GFP-P^mIDR2^) inhibited spherical droplet formation, underscoring the importance of IDR2 in GFP-P condensate formation (fig. S2). These results validate the occurrence of LLPS of the RSMV P protein in vitro.

The ability of BFP-P granules to recruit N-GFP protein in vivo (Fig. 1H) prompted an investigation into the potential for RSMV P–formed droplets to concentrate RSMV N protein and genomic RNAs in vitro. Purified mCherry-N with free GFP did not undergo LLPS; however, incubation with GFP-P resulted in the gradual incorporation of mCherry-N into GFP-P–formed droplets (Fig. 2F). Subsequent experiments using a 227-nucleotide Cy5-labeled 5′ trailer of the RSMV genome demonstrated that both mCherry-N and Cy5-Trailer were incorporated into GFP-P droplets, whereas they remained evenly distributed when incubated with GFP alone (Fig. 2G). These findings collectively indicate that RSMV P–formed liquid droplets have the capacity to encapsulate RSMV N protein and the 5′ trailer of the RSMV genome in vitro.

### RSMV P interacts with a key component of the COP I, OsARF1C

**Fig. 3.**
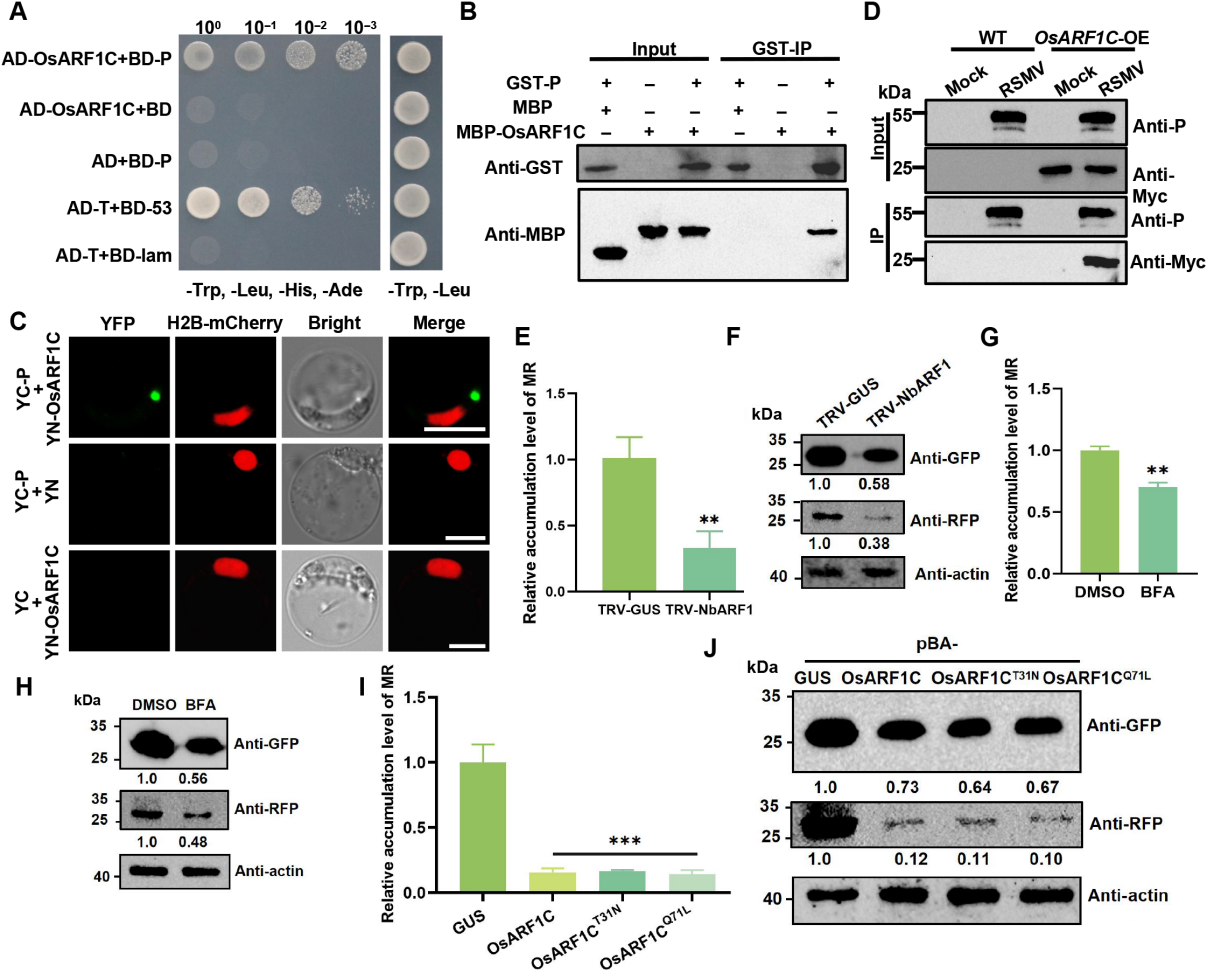
Interaction of OsARF1C with P and its role in viral MR replication. (**A**) The interaction between P and OsARF1C was assessed using a Y2H assay. Yeast strain Y2HGold cultures, cotransformed with the indicated plasmids, were grown on SD-Trp-Leu-His-Ade selection medium. (**B**) In vitro glutathione *S*-transferase (GST) pull-down assays were conducted to explore the interaction between P and OsARF1C. (**C**) BiFC assay validated the interaction in rice protoplasts. Scale bars, 10 μm. (**D**) Co-IP assays confirmed the interaction between OsARF1C and RSMV P. WT and OsARF1C-OE plants were mock- or RSMV-inoculated and were harvested at 15 dpi. Total proteins were immunoprecipitated with anti-P beads. Input and IP proteins were analyzed by protein gel blot analysis with anti-Myc and anti-P antibodies. (**E**, **G**, and **I**) RT-qPCR analysis of minigenome RNA replication in the MR system at 6 dpi with various treatments. *NbPP2A* was used as an internal control gene. The values represent means ± SD (*n* = 4). ** and *** indicate highly significant difference (*P* < 0.01 and *P* < 0.001, respectively) based on the Tukey-Kramer post hoc test. (**F**, **H**, and **J**) Accumulation of GFP and RFP in the MR-inoculated *N. benthamiana* leaves at 6 dpi was measured via Western blotting, with relative protein band intensities normalized against that of actin.

A yeast two-hybrid (Y2H) screening was used to find host factors implicated in RSMV replication by interacting with the P protein. Considering the ER localization of the P protein, OsARF1C emerged as a potential interacting partner, as it plays a crucial role in the COP I vesicle transport pathway (Fig. 3A). This interaction was validated through protein affinity isolation (Fig. 3B). Bimolecular fluorescence complementation (BiFC) further demonstrated that the P protein interacts with OsARF1C in the cytoplasm, forming punctate granules (Fig. 3C). Subsequently, we generated OsARF1C overexpression transgenic lines (*OsARF1C-OE*) with a Myc tag fused to its C terminus. Coimmunoprecipitation (co-IP) assays indicated that the RSMV P protein was able to precipitate with OsARF1C-Myc following RSMV infection (Fig. 3D), confirming the existence of the OsARF1C-P protein complex in planta.

The rice genome contains six ARF1 members: OsARF1A, OsARF1B, OsARF1C, OsARF1D, OsARF1E, and OsARF1F (*50*). A comparative analysis of protein sequences among these six OsARF1 variants revealed that all ARF1 proteins consist of 181 amino acids and demonstrate remarkable sequence conservation (fig. S3A). BiFC assays indicated that the RSMV P protein interacts with all OsARF1 family members (fig. S3B). Consequently, OsARF1C was selected as a representative for further functional investigations. The transcription levels of OsARF1 remained unchanged during RSMV infection compared to healthy plants (fig. S3C).

### The activity and homeostasis of OsARF1 are critical for RSMV infection

To investigate the impact of ARF1 on RSMV infections in plants, we used tobacco rattle virus (TRV)–based virus-induced gene silencing to down-regulate *ARF1* in *N. benthamiana* plants (fig. S3D). Subsequently, the silenced plants were inoculated with the RSMV MR system (fig. S3E) (*48*). A notable reduction in both genomic RNA and reporter protein levels of MR was observed in NbARF1-silenced plants (Fig. 3, E and F), indicating the critical role of ARF1 in RSMV MR replication.

Brefeldin A (BFA) inhibits protein secretion from the Golgi apparatus to the ER by disrupting ARF protein nucleotide exchange reactions (*51*). We tested the effect of BFA and observed that its treatment notably reduced OsARF1C expression and impaired colocalization between the viral P protein and OsARF1C (fig. S3F). Moreover, BFA treatment markedly decreased BFP-P granule size compared to the control (fig. S3, G and H). Then, *N. benthamiana* leaves were infected with RSMV MR for 6 days, followed by a 24-hour treatment with BFA. Subsequent reverse transcription quantitative polymerase chain reaction (RT-qPCR) analysis demonstrated a significant reduction in MR RNA accumulation (Fig. 3G). Simultaneously, the levels of GFP and red fluorescent protein (RFP) decreased (Fig. 3H). These findings suggest that the BFA-mediated inhibition of ARF1 function adversely affects the replication of RSMV MR.

ARFs function as molecular switches, transitioning between an inert state bound to guanosine diphosphate (GDP) and an activated state bound to guanosine triphosphate (GTP) (*52*). We generated a GDP-locked mutant of OsARF1C, designated OsARF1C^T31N^, in which the threonine at position 31 was substituted with asparagine, and a GTP-locked mutant of OsARF1C, denoted OsARF1C^Q71L^, where the glutamine at position 71 was replaced with leucine (fig. S3A). BiFC analysis revealed that both mutants maintained the interactions with P proteins (fig. S4A). Overexpression of OsARF1C^T31N^ or OsARF1C^Q71L^ in *N. benthamiana* significantly reduced MR RNA accumulation (Fig. 3I). Western blot analysis corroborated the decreased levels of GFP and RFP in coinfiltrated tissues (Fig. 3J). Wild-type (WT) OsARF1C overexpression also diminished RNA and reporter protein levels of the RSMV MR, suggesting a potential role for ARF1 in viral disease resistance (Fig. 3, I and J).

**Fig. 4.**
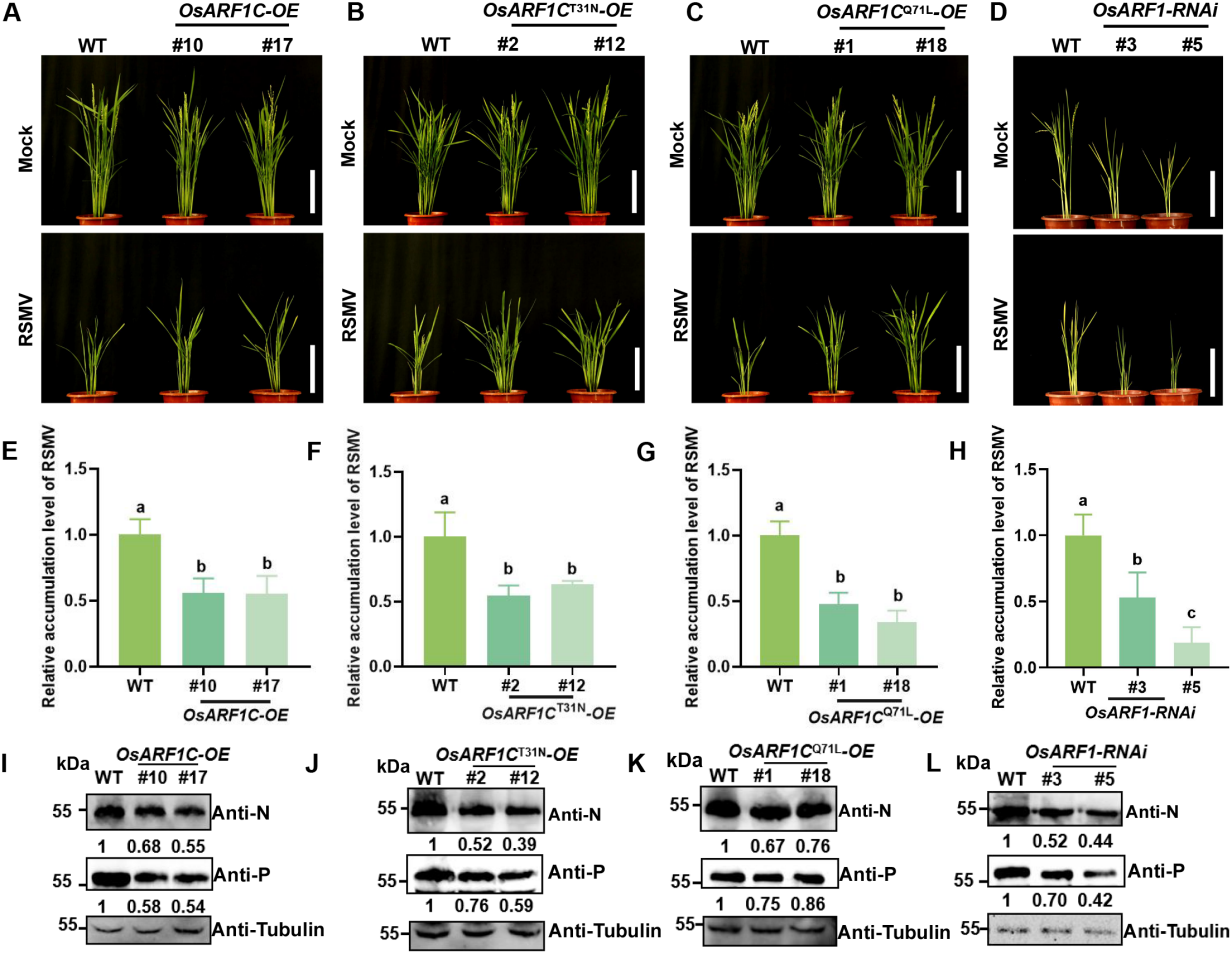
The role of OsARF1C in RSMV infection. (**A** to **C**) Phenotypes of mock- and RSMV-inoculated WT, *OsARF1C-OE*, *OsARF1C*^T31N^*-OE*, and *OsARF1C*^Q71L^*-OE* transgenic rice plants at 35 dpi. Scale bars, 15 cm. (**D**) Phenotypes of mock- and RSMV-inoculated WT and *OsARF1-RNAi* transgenic rice plants at 20 dpi. Scale bars, 15 cm. (**E** to **H**) Accumulation of RSMV RNA was assessed in RSMV-inoculated WT, *OsARF1C-OE*, *OsARF1C*^T31N^*-OE*, and *OsARF1C*^Q71L^*-OE* transgenic rice plants at 35 dpi, as well as in RSMV-inoculated WT and *OsARF1-RNAi* transgenic rice plants at 20 dpi, by RT-qPCR. Shown values represent the means ± SD (*n* = 4). Different letters indicate significant differences (*P* < 0.05) based on the Tukey-Kramer post hoc test. (**I** to **L**) Accumulation of RSMV N and P proteins was assessed in RSMV-inoculated WT, *OsARF1C-OE*, *OsARF1C*^T31N^*-OE*, and *OsARF1C*^Q71L^*-OE* transgenic rice plants at 35 dpi, as well as in RSMV-inoculated WT and *OsARF1-RNAi* transgenic rice plants at 20 dpi, using Western blot assays. The relative intensity of protein bands was normalized against tubulin.

In a manner consistent with the *OsARF1C-OE*, we generated transgenic rice lines that overexpressed OsARF1C^T31N^ and OsARF1C^Q71L^, respectively. The overexpressing plants were then inoculated with RSMV. Compared to WT plants, these transgenic lines exhibited milder disease symptoms (Fig. 4, A to C) and showed reduced levels of viral RNA (Fig. 4, E to G) and N and P proteins (Fig. 4, I to K). In addition, we developed OsARF1 RNA interference (RNAi) lines targeting conserved sequences of six *OsARF1* genes. These RNAi lines demonstrated stunted growth and ultimately died (Fig. 4D), highlighting the critical role of ARF1 in plant development. Upon RSMV infection, the RNAi lines displayed attenuated viral phenotypes (Fig. 4D), with decreased levels of viral RNA and proteins (Fig. 4, H and L). Collectively, these findings indicate that the activity and homeostasis of OsARF1 are essential for RSMV replication.

### RSMV P recruits OsPI4KB into granules via OsARF1C

**Fig. 5.**
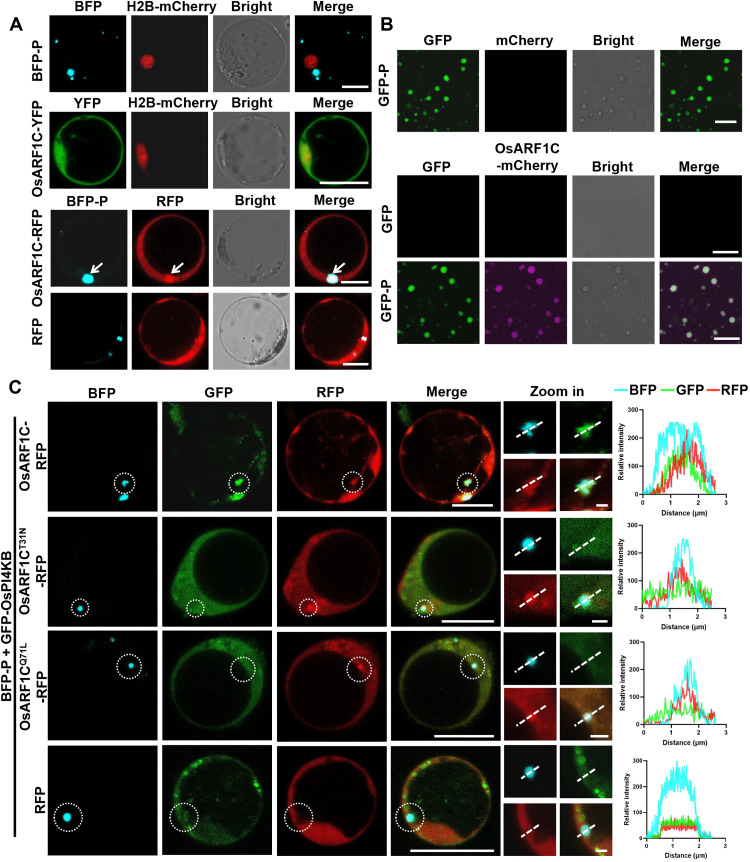
The P protein of RSMV hijacks OsPI4KB into its granules via OsARF1C. (**A**) Confocal images showing the subcellular localization of P and OsARF1C in rice protoplasts. Scale bars, 10 μm. (**B**) Confocal images demonstrating the incorporation of OsARF1C-mCherry into GFP-P droplets. Free GFP was unable to form droplets to recruit OsARF1C-mCherry. Scale bars, 20 μm. (**C**) Confocal images illustrating the colocalization of BFP-P, GFP-PI4KB, and OsARF1C-RFP in rice protoplasts. Scale bars, 10 μm. Zoomed area scale bars, 1 μm. The figures on the right panel represent the normalized fluorescence intensities of the BFP, GFP, and RFP channels along the dashed white lines in the merged images.

To elucidate the interaction mechanism between OsARF1C and the RSMV P protein, we investigated their subcellular localization rice protoplasts. OsARF1C–yellow fluorescent protein (YFP) localized to the cytoplasm, whereas BFP-P formed punctate granules. Coexpression revealed partial colocalization, with a subset of OsARF1C-YFP overlapping with the punctate granules (Fig. 5A). Furthermore, both the GDP-locked mutant and the GTP-locked mutant can be recruited into punctate granules formed by BFP-P (fig. S4B). In vitro investigations demonstrated that purified OsARF1C-mCherry did not exhibit LLPS with free GFP. However, coincubation with GFP-P resulted in the integration of OsARF1C-mCherry into droplets formed by the P protein (Fig. 5B). These findings suggest that the P protein has the capacity to recruit OsARF1C.

ARF1 is crucial for protein and lipid transport in mammalian cells, specifically mediating PI4P synthesis through its interaction with PI4KB. Here, ARF1 facilitates and stimulates the generation of PI4P (*27*). BiFC assays revealed an interaction between OsARF1C and OsPI4KB (fig. S4C). Subcellular localization studies demonstrated colocalization of OsPI4KB with OsARF1C and its mutant forms, OsARF1C^T31N^ and OsARF1C^Q71L^ (fig. S4D). Further analysis of colocalization with RSMV P protein showed that the P protein can recruit both OsARF1 and OsPI4KB into punctate granules. However, when OsARF1C^T31N^ was present, the P protein could recruit OsARF1 but not OsPI4KB. In contrast, with OsARF1^Q71L^, neither of the proteins were recruited, and the fluorescence intensity analysis confirmed these observations (Fig. 5C). These results indicate that the recruitment of OsPI4KB by RSMV P protein is dependent on the activity of OsARF1C.

### OsPI4KB generates PI4P to facilitate RSMV infection

**Fig. 6.**
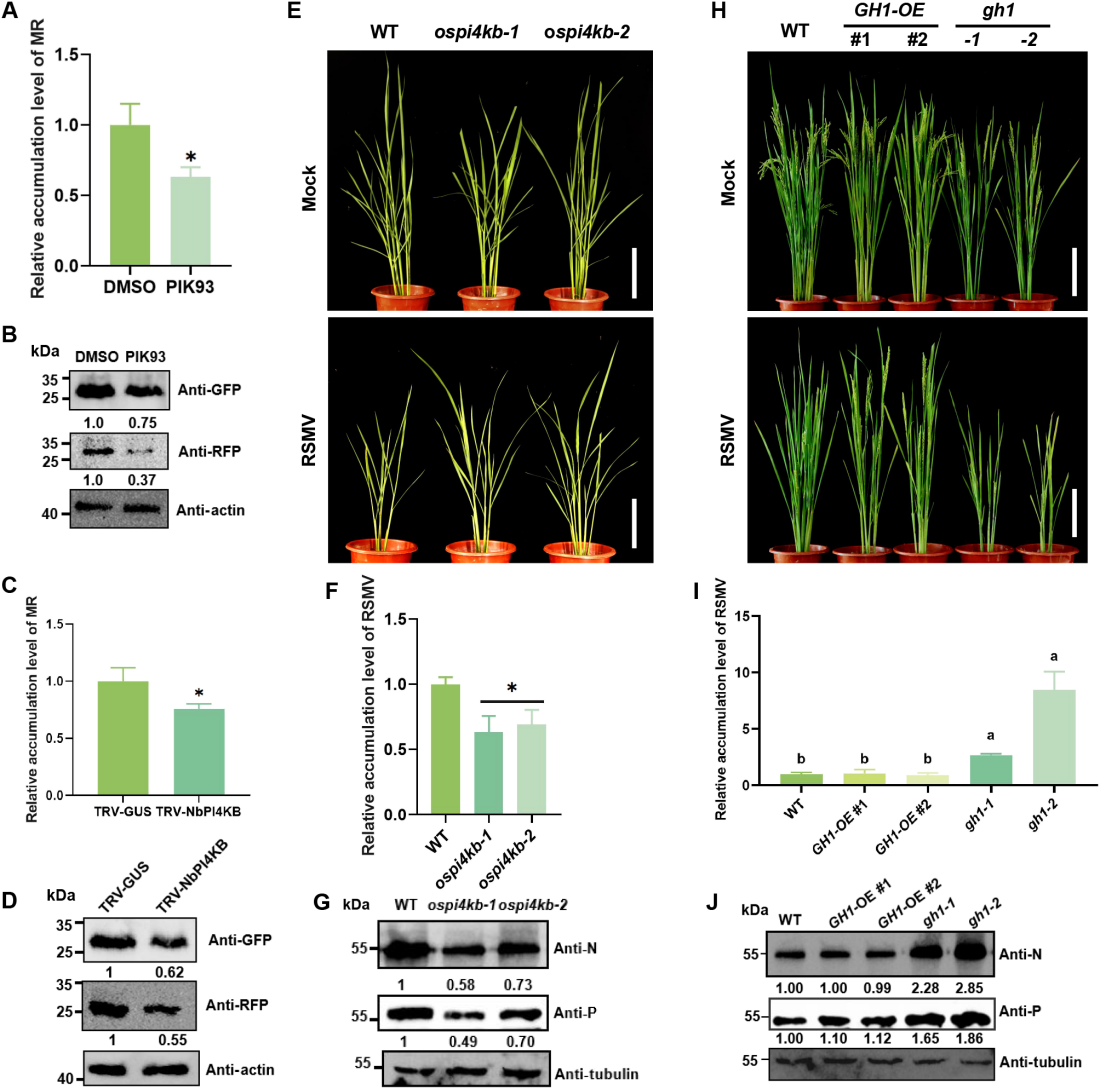
OsPI4KB generates PI4P to facilitate RSMV infection. (**A**) RT-qPCR analysis of minigenome RNA replication in the MR system at 6 dpi with PIK93 treatments. *NbPP2A* served as an internal control gene. The values shown are the means ± SD (*n* = 4). Student’s *t* test was used for analyses, and * indicates that the difference between the data is significant (*P* < 0.05). (**B** and **D**) Accumulation of GFP and RFP in the MR-inoculated *N. benthamiana* leaves at 6 dpi was determined by Western blot assay. The relative protein band intensity was normalized against that of actin. (**C**) RT-qPCR analysis of minigenome RNA replication at 6 dpi in TRV-induced gene silenced *N. benthamiana* leaves, using *NbPP2A* as an internal control gene. The values shown are the means ± SD (*n* = 4). Student’s *t* test was used for analyses, and * indicates significant differences (*P* < 0.05). (**E** and **H**) Phenotypes of mock-inoculated and RSMV-inoculated WT, *ospi4kb*, *GH1-OE*, and *gh1* transgenic rice plants at 35 dpi. Scale bars, 15 cm. (**F** and **I**) Accumulation of RSMV RNA in WT, *ospi4kb*, *GH1-OE*, and *gh1* transgenic rice plants inoculated with RSMV at 35 dpi was determined by RT-qPCR. The values shown are the means ± SD (*n* = 4). Different letters or * indicate significant differences (*P* < 0.05) based on the Tukey-Kramer post hoc test. (**G** and **J**) Accumulation of RSMV N and P proteins in *ospi4kb*, *GH1-OE*, and *gh1* transgenic rice plants at 35 dpi was determined by Western blot assay. The relative protein band intensity was normalized against that of tubulin.

The function of OsPI4KB in RSMV replication was investigated using the RSMV MR system in *N. benthamiana* under various experimental conditions. PIK93, a PI4K inhibitor (*53*), was applied to RSMV MR–infected tobacco leaves at 6 days postinoculation (dpi). RT-qPCR analysis performed 24 hours later revealed a significant reduction in MR RNA accumulation upon the application of PIK93 (Fig. 6A), which was associated with corresponding decreases in GFP and RFP protein levels (Fig. 6B). Subsequently, the TRV system was used to silence *NbPI4KB* expression (fig. S5A). When challenged with the RSMV MR system, *NbPI4KB*-silenced plants exhibited impaired viral replication, characterized by diminished levels of MR RNA and GFP/RFP proteins compared to β-glucuronidase (GUS)-silenced control plants (Fig. 6, C and D).

In addition, CRISPR-Cas9–generated *ospi4kb* mutants (fig. S5B) were subjected to RSMV infection. Comparative analysis revealed that *ospi4kb* mutant plants exhibited milder dwarfism upon infection (Fig. 6E), accompanied by decreased levels of RSMV RNA and N/P protein (Fig. 6, F and G). The rice *GH1* (GRAIN NUMBER AND PLANT HEIGHT1), a PI4P phosphohydrolase with a suppressor of actin (SAC) domain, plays a negative regulatory role in PI4P levels (*54*). While *gh1* mutants with increased PI4P exhibit pronounced disease symptoms, plants overexpressing GH1 (*GH1-OE*) displayed symptoms comparable to those of WT plants (Fig. 6H). The *gh1* mutants showed elevated levels of RSMV RNA and N/P protein, in contrast to the similar levels observed in *GH1-OE* and WT plants (Fig. 6, I and J). These findings suggest that PI4P facilitates RSMV infection.

### PI4P accumulates within the RSMV VFs

**Fig. 7.**
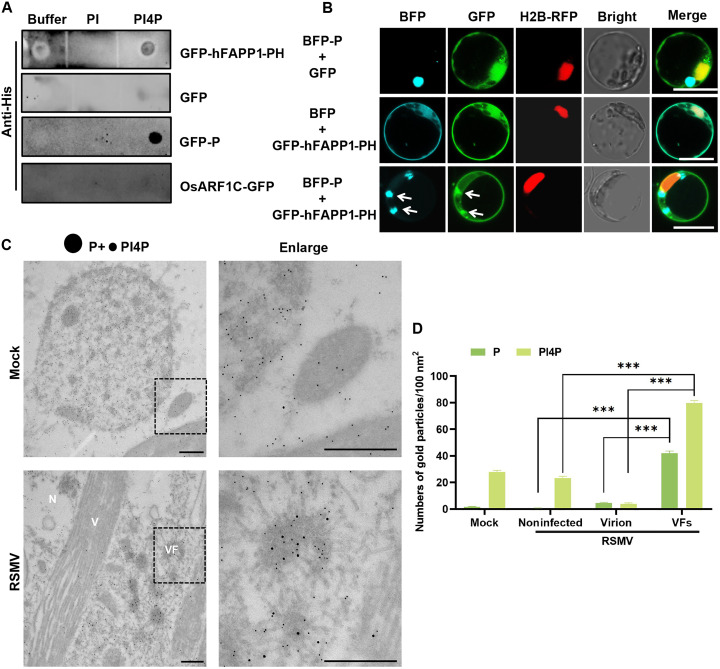
Enrichment of P-binding PI4P in VFs. (**A**) Analysis of the interaction between RSMV P and PI4P using a lipid binding assay. PI was included as a control, where the binding of hFAPP1-PH to PI4P served as a positive control. (**B**) Confocal microscopy images demonstrate the colocalization of P and PI4P in rice protoplasts, with GFP-hFAPP1-PH indicating the PI4P distribution. Scale bars, 10 μm. (**C**) Immunosorbent electron microscopy analysis showing the subcellular localization of P and PI4P in noninfected versus RSMV-infected rice stem cells at 30 dpi, with P and PI4P labeled using 10- and 18-nm gold particles, respectively. Scale bars, 500 nm. N, noninfected area; V, virion. (**D**) Quantification of P protein and PI4P colloidal gold particles in mock, noninfected, virion, and VF regions. Gold particle counts were performed in 30 cells. *** indicates a highly significant difference (*P* < 0.001) based on the Tukey-Kramer post hoc test.

We postulate that RSMV VFs are characterized by a high abundance of PI4P, as indicated by the recruitment of OsPI4KB. To investigate the potential binding of the P protein to PI4P, we used the molecular docking performed by Chai-1, indicating the interaction between P and PI4P (fig. S6). Furthermore, we performed a lipid-protein binding assay. Purified GFP, GFP-P, and OsARF1C-GFP were incubated on strips containing PI4P, PI, and buffer, with the PI4P biosensor (GFP-human four-phosphate-adaptor protein 1-pleckstrin homology domain, GFP-hFAPP1-PH) serving as a positive control (*55**,* *56*). Results demonstrated that GFP-P selectively bound to PI4P, exhibiting a higher binding capacity than the GFP-hFAPP1-PH control (Fig. 7A). In addition, we conducted an in vivo analysis of PI4P and RSMV P colocalization, using GFP-hFAPP1-PH as a PI4P marker in plant cells. Our findings indicated PI4P accumulation within P protein granules in rice protoplasts (Fig. 7B).

To investigate the distribution of PI4P in rice cells infected with RSMV, we used immunoelectron microscopy on samples from both mock-infected and RSMV-infected plants. Using gold particles of two distinct sizes to label the P protein and PI4P, we observed that while PI4P was uniformly distributed in mock-infected cells, RSMV-infected cells exhibited significant enrichment of gold particles within VFs. This enrichment indicates substantial aggregation of both P protein and PI4P within these structures. Conversely, few gold particles were detected in regions resembling virions, and virus-free areas retained a uniform PI4P distribution similar to that in mock-infected cells (Fig. 7, C and D). These findings provide conclusive evidence of substantial PI4P accumulation within RSMV-induced VFs.

### PI4P enhances viral replication by influencing the P granules size

**Fig. 8.**
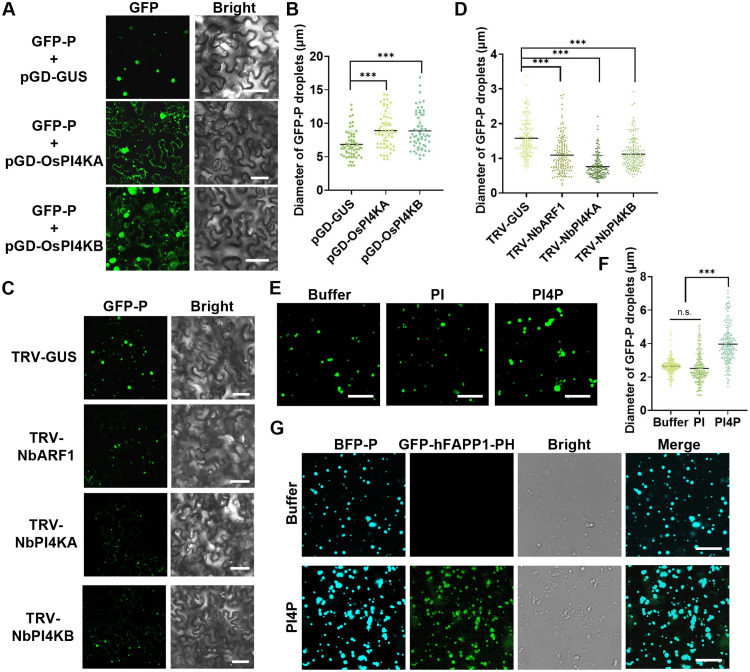
Modulation of P granule size by PI4P in vivo and in vitro. (**A**) Confocal images depicting GFP-P granules following coinfiltration of GFP-P with PI4KA/B in *N. benthamiana* leaves. Scale bars, 10 μm. (**B**) Statistical analysis of GFP-P granule diameter in panel (A), with at least 50 GFP-P granules measured. *** denoting a highly significant difference (*P* < 0.001) based on the Tukey-Kramer post hoc test. (**C**) Confocal images illustrating GFP-P granules in *N. benthamiana* plants treated with TRV-GUS, TRV-NbARF1, TRV-NbPI4KA, and TRV-NbPI4KB, respectively. Scale bars, 10 μm. (**D**) Statistical analysis of GFP-P granule diameter in panel (C), with a minimum of 150 GFP-P granules measured. *** indicates a highly significant difference (*P* < 0.001) based on the Tukey-Kramer post hoc test. (**E**) Confocal images of GFP-P droplets in buffers with PI or PI4P, respectively, with buffer concentration at 5 μM in 100 mM NaCl. Scale bars, 10 μm. (**F**) Statistical analysis of GFP-P droplet diameter in panel (E), involving at least 180 GFP-P droplets measured. *** indicating a highly significant difference (*P* < 0.001) based on the Tukey-Kramer post hoc test. n.s., not significant. (**G**) Confocal images revealing the distribution of PI4P in GFP-P droplets, at a concentration of 10 μM in 100 mM NaCl. GFP-hFAPP1-PH indicates the location of PI4P. Scale bars, 10 μm.

To investigate how PI4P regulates the formation of RSMV VFs, we transiently overexpressed OsPI4KA and OsPI4KB in *N. benthamiana* to elevate cellular PI4P levels (fig. S7A). Confocal microscopy revealed that GFP-P formed larger granules when coexpressed with either OsPI4KA or OsPI4KB compared to the controls (Fig. 8A). Statistical analysis of granule diameters supported the hypothesis that increased PI4P levels promote LLPS of the RSMV P protein (Fig. 8B). Furthermore, we used the TRV system to silence PI4K or ARF1 to inhibit PI4P synthesis and observed GFP-P granule formation in *N. benthamiana* cells (fig. S7, B to D). The results indicated that silencing PI4K or ARF1 significantly reduced GFP-P granule size compared to controls (Fig. 8, C and D), suggesting that PI4P positively regulates RSMV P protein LLPS in vivo.

We next investigated the influence of PI4P on the LLPS of the P protein in vitro. Purified GFP-P protein supplemented with PI4P, but not with PI, induced larger spherical LLPS droplets compared to the buffer control (Fig. 8E), resulting in a significant increase in droplet diameter (Fig. 8F). To verify the spatial arrangement of PI4P, we used GFP-hFAPP1-PH, a PI4P-specific probe, alongside BFP-P proteins. Control trials lacking PI4P exhibited no GFP emission within the BFP-P droplets. However, upon the introduction of PI4P, droplet enlargement occurred, accompanied by overlapping fluorescence signals from both GFP and BFP (Fig. 8G). This observation suggests a homogeneous distribution of PI4P and enhanced LLPS of the P protein.

## DISCUSSION

Biomolecular condensates formed through LLPS not only regulate diverse cellular functions but also play an important role in the formation of VFs/inclusion bodies/viroplasms during virus infection. For instance, the N protein of SARS-CoV-2 undergoes LLPS to sequester components of the viral replication machinery (*10*). Similarly, the RdRp of HuNoV forms distinct condensates exhibiting LLPS properties, which include sustained polymerase activity and the recruitment of crucial viral replication elements (*13*). In the case of plant viruses, although not directly implicated in VF formation, the movement protein p26 of pea enation mosaic virus 2 has been observed to undergo phase separation and interact with cellular factors, thereby modulating viral replication (*57*). In addition, the P protein encoded by BYSMV demonstrates LLPS and contributes to viral replication (*22*, *58*). Here, we observed numerous membraneless spherical regions in rice cells infected with RSMV through electron microscopy, with assembled viral RNPs found to radiate outward, indicating that these spherical regions are VFs of RSMV. Further immunoelectron microscopy revealed that the P protein is predominantly localized within the VFs, while its concentration is relatively low in fully assembled RNPs or mature viral particles, suggesting that the P protein is a core component of RSMV VFs. We have also confirmed the LLPS characteristics of the P protein in both in vitro and in vivo experiments, demonstrating its ability to recruit key components for viral replication, including the N protein and viral RNA. These findings underscore the role of the P protein in the construction of RSMV VFs through LLPS.

Previous studies on rhabdoviruses infecting animals and plants, such as RABV (*18*) and BYSMV (*22*), have indicated that their P proteins contain IDRs capable of undergoing LLPS. This LLPS process in the P protein plays a crucial role in recruiting viral RNAs, viral proteins, and host factors necessary for viral replication, effectively acting as a hub for efficient viral replication. Furthermore, the membraneless organelles formed through phase separation of the P protein may serve to exclude proteins with antiviral properties from the site of viral replication.

The occurrence of protein LLPS is regulated by various factors, including protein concentration, salt concentration, pH, temperature, and solution crowding (*1*, *3*). To our knowledge, this is the first report demonstrating the promoting effect of lipids on protein phase separation. We demonstrated that the addition of lipids to the RSMV P protein in vitro LLPS system revealed that PI4P, rather than PI, facilitates the formation and enlargement of P protein droplets. This finding broadens our understanding of the regulatory factors governing protein LLPS and suggests a crucial role for PIPs in viral replication. However, it remains unclear how PIPs regulate the LLPS of viral replication–related proteins and whether PIPs exert a similar promoting effect on the LLPS of other nonviral proteins, warranting further investigation. Recent studies underscore the importance of phase separation in plant disease resistance. Our research demonstrates that PI4P lipids regulate protein phase separation, presenting a potential target for developing disease-resistant crops. However, direct lipid pathway manipulation may compromise plant growth due to their intricate functions. Precise CRISPR editing of the rice *RBL1* (RESISTANCE TO BLAST1) gene, which modulates PI(4,5)P2, provides a strategic approach to balancing disease resistance and developmental processes (*59*). Complementary chemical interventions, such as the small molecule Z9, effectively suppress viral nucleoprotein condensate formation, substantially inhibiting viral infection (*20*). These advances translate phase separation insights into sophisticated, sustainable strategies for crop protection.

PIPs play a critical role in viral replication; however, viruses and their proteins use diverse mechanisms to hijack PIPs. The first mechanism involves the direct binding of PIPs by membrane-localized viral proteins. For instance, the H7 protein of the vaccinia virus can directly bind to PI3P and PI4P to facilitate viral infection (*60*). Similarly, the group-specific antigen protein (Gag) of HIV directly interacts with PI(4,5)P2 (*61*). Moreover, the P10 protein of rice black streak dwarf virus can bind to PI(3,5)P2 within its insect host, inhibiting antiviral autophagy and promoting viral replication (*62*). The second mechanism entails viral proteins recruiting PI kinases to synthesize specific PIPs necessary for viral propagation. For example, the nonstructural protein NS5A of hepatitis C virus (HCV) interacts directly with PI4KA, and mutations at the interaction site notably inhibit HCV replication (*38*). In HCV-infected cells, there is a notable increase in PI4P levels without a corresponding elevation in PI4KA transcription, indicating that HCV hijacks PI4KA to replication sites, using its kinase activity to generate more PI4P, which is conducive to viral replication (*38*). Similarly, human RSV recruits PI4KB to its replication complex via the N protein to generate PI4P, thereby enhancing viral replication (*28*). In addition, the replication protein p33 of TBSV interacts directly with the host Vps34, a type of phosphatidylinositol 3-kinase (PI3K), establishing a viral replication microenvironment enriched with PI3P that facilitates viral replication (*63*). The third mechanism, akin to the findings of this study, involves viral proteins recruiting cellular trafficking proteins to indirectly recruit PI kinases for localized PIP synthesis, thereby promoting viral replication. TBSV can also exploit the retromer complex proteins via its replication protein p33, facilitating the transport of phosphatidylserine decarboxylase Psd2, PI3K, and PI4K through the retromer trafficking pathway to the viral replication complex (*63*). Consequently, a complex enriched with phosphatidylethanolamine, PI3P, and PI4P is formed, ultimately enhancing viral replication (*29*).

In eukaryotic cells, the membrane-bound ARF1 protein plays a crucial role in the replication of various RNA viruses. This study confirmed the interaction between RSMV P protein and OsARF1, which leads to the indirect recruitment of OsPI4KB for the formation of RSMV VFs. Both silencing and overexpressing OsARF1 were found to be detrimental to RSMV replication. The inhibition of OsARF1 expression disrupts the recruitment of the P protein to PI4KB, thereby inhibiting viral infection. In addition, ARF1 not only functions as a key protein in COP I vesicle transport but also plays a critical role in plant defense (*64*). Overexpression of ARF1 in tobacco and rice plants enhances their resistance, leading to a notable increase in the expression of resistance-related genes, up-regulation of salicylic acid accumulation, and induction of cell death (*65*, *66*). Furthermore, the overexpression of ARF1 in both rice and tobacco plants substantially improves resistance against pathogens, which may explain the observed inhibition of RSMV infection in rice plants overexpressing OsARF1C (*64*). On the other hand, the stable transport of PI4K by ARF1 relies on the switching of its activity states (associated with GDP or GTP binding). The inactive mutant (OsARF1C^T31N^) fails to transport PI4K, while the constitutively active mutant (OsARF1C^Q71L^) disrupts Golgi and ER integrity (*52*), also leading to decreased PI4K recruitment ability. Our findings convincingly demonstrate that both mutants of OsARF1C lose the capacity to facilitate the indirect recruitment of OsPI4KB by P protein, resulting in the inhibition of RSMV infection. Moreover, silencing ARF1 disrupts intracellular vesicular trafficking, hindering the transportation of cargo proteins and small molecules within cells, thereby impeding plant development and potentially leading to premature death (*65*). The study also observed stunted growth in *OsARF1-RNAi* plants, with diminished plant vigor possibly compromising viral replication. Therefore, maintaining the homeostasis of ARF1-like vesicle transport proteins in host cells is crucial for successful infection of RSMV.

PI4P is dynamically generated in vivo primarily by phosphorylating PIP through PI4K. Subsequently, Sac1 phosphatase recognizes and dephosphorylates PI4P back to PIP. In rice, GH1 functions as the Sac1 homolog, negatively regulating PI4P (*54*). The *gh1* mutant rice plants showed significantly increased RSMV accumulation, indicating a role for PI4P in RSMV replication. However, overexpressing *GH1* did not notably impede viral replication. There are two possibilities to consider. First, the basal level of RSMV infection does not rely on a high accumulation of cytoplasmic PI4P. Even with *GH1* overexpression leading to a decrease in cellular PI4P content, the remaining PI4P is adequate for supporting the establishment of RSMV VFs and viral infection. Second, the P protein of RSMV indirectly recruits PI4KB to onsite synthesis of PI4P at the VFs to promote viral replication. The LLPS of the P protein aids in sequestering PI4P within the VFs. Therefore, despite *GH1* overexpression affecting only free PI4P in the cell, it does not disrupt the PI4P within the VFs, thus leaving RSMV infection unaffected.

**Fig. 9.**
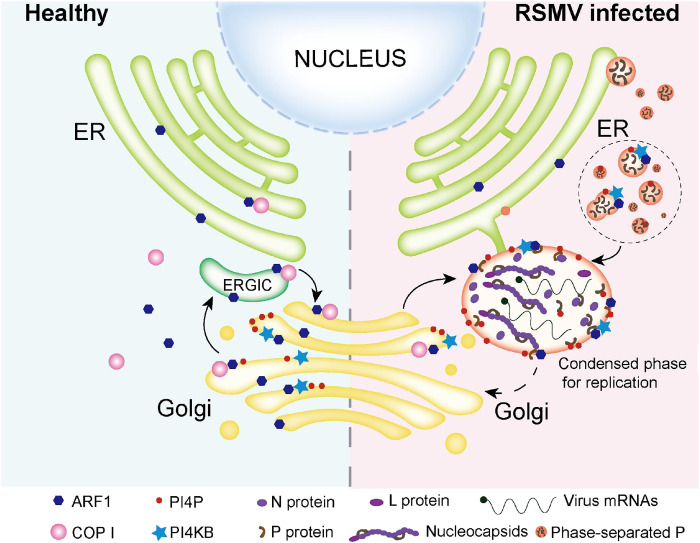
A proposed model illustrating how RSMV hijacks host trafficking and lipid synthesis for VF assembly. In healthy rice cells (left), ARF1 and COP I coats facilitate trafficking to the ERGIC and Golgi/TGN compartments, with PI4KB enzymes recruited to Golgi membranes by OsARF1 to produce PI4P lipids. In RSMV-infected rice cells (right), the P protein recruits OsARF1-PI4KB to establish a PI4P-enriched RSMV VF, binding PI4P to induce P LLPS droplets that expand the VF area, thereby enhancing RSMV replication efficiency.

In summary, our results demonstrate that the RSMV P protein directly interacts with OsARF1C, a crucial component of the COP I vesicle transport pathway essential for viral replication. This interaction indirectly recruits OsARF1C’s partner, OsPI4KB, which synthesizes PI4P and binds to the P protein. Meanwhile, the P protein modulates its aggregates and LLPS droplets via PI4P, leading to the formation of a larger replication site for the virus (Fig. 9). These findings provide potential targets for effectively controlling similar viruses, offering promising strategies for disease prevention and control.

## MATERIALS AND METHODS

### Plant materials and growth conditions

The rice plants used in this study for overexpression, RNAi, and the mutant lines of *OsARF1C* and *OsPI4KB* were of the cultivar Zhonghua 11 background. In contrast, the transgenic lines of RSMV P were derived from the cultivar Nipponbare background, while the overexpression and mutant lines associated with *GH1* were based on the cultivar GC background. Transgenic rice plants overexpressing *OsARF1C-OE*, *OsARF1C*^T31N^*-OE*, *OsARF1C*^Q71L^*-OE*, and *OsARF1-RNAi* were generated in this study. The *ospi4kb* mutant lines were developed using CRISPR-Cas9 technology at the Biogle Genome Editing Center (Jiangsu, China). Seeds of the RSMV P transgenic lines were obtained as described previously (*47*). In addition, seeds for *GH1-OE* and *gh1* mutant lines were provided by H. Lin at the Institute for Plant Physiology and Ecology, Chinese Academy of Sciences, Shanghai, China (*54*). All rice plants were grown in a greenhouse maintained at 28°C with 60 ± 5% relative humidity and exposure to natural sunlight. *N. benthamiana* plants, including transgenic lines expressing RFP–histone 2B (H2B) as a nuclear marker, were cultivated in growth chambers at 25°C, under a photoperiod of 16 hours of light followed by 8 hours of darkness.

### RSMV inoculation

RSMV was maintained through serial transmission by leafhoppers (*Recilia dorsalis*) within rice plants, as previously described (*46*). Two-week-old seedlings were exposed to either RSMV-carrying or virus-free (mock) leafhoppers, with two to three insects per plant for a duration of 3 days. Following this exposure, the presence of the virus in RSMV-infected rice plants or in viruliferous *R. dorsalis* was confirmed using RT-qPCR or reverse transcription-recombinase aided amplification (RT-RAA)–CRISPR-Cas12a visual detection as previously described (*47*, *67*).

### Plasmid constructions

All primers used for cloning experiments are listed in table S1. The coding sequences of the RSMV genes were amplified using cDNA derived from RSMV-infected rice plants as a template. The coding sequence of hFAPP1-PH from humans was amplified using the cDNA of human tissues as a template. The RSMV MR system, which includes pRSMV-MR, pGD-NPL, pGD-VSRs, pCambia1300-GFP-P, and pCambia1300-N-GFP, was previously constructed (*48*). All genes from rice and tobacco were amplified using cDNA extracted from the respective plants. Point mutants of OsARF1C were generated using the QuickChange mutagenesis method.

To obtain the plasmids pGADT7-AD-OsARF1C, pGBKT7-BD-P, pGEXT-P, pMBP-OsARF1C, pRHV-cMYC-OsARF1C/OsARF1C^T31N^/OsARF1C^Q71L^, pTRV2-NbARF1/NbPI4KB/NbPI4KA, pCambia-1300-GFP-hFAPP1-PH, pCV-nYFP-OsARF1B/D/F, and pCV-cYFP-OsPI4KB, the full-length coding sequences of the targeted genes were cloned and individually inserted into the vectors using the In-Fusion cloning kit (Vazyme Biotech, C112). In addition, to obtain the plasmids pGD-BFP-P, pGD-OsARF1C/OsARF1C^T31N^/OsARF1-C^Q71L^-RFP, pGD-GFP-OsPI4KB, pET28a-GFP-hFAPP1-PH, pET28a-GFP-P, and pET28a-OsARF1C-GFP, the full-length coding sequences of the targeted genes were cloned and overlapped with GFP, BFP, or RFP sequences and subsequently inserted into the vector using the In-Fusion cloning kit (Vazyme Biotech, C112).

To obtain pEarleyGate202-YC-P, pEarleyGate202-YN-OsARF1C, pBA-35S-FlagMyc4-OsARF1C/OsARF1C^T31N^/OsARF1C^Q71L^, and pEarleyGate102-OsARF1C-YFP, the full-length coding sequences of P, OsARF1C, OsARF1C^T31N^, or OsARF1C^Q71L^ were individually cloned into the pENTR/D-TOPO vector (Thermo Fisher Scientific, K240020). Subsequently, the cloned sequences were transferred into the corresponding destination vectors using a Gateway LR reaction kit in accordance with the manufacturer’s instructions (Thermo Fisher Scientific, K240020).

### RNA extraction and RT-qPCR

Total RNA was extracted from rice tissue samples using the Total RNA Extraction Reagent (Vazyme Biotech, R401). To detect relative gene expression levels in rice and *N. benthamiana*, we synthesized cDNA from the isolated total RNA using an oligo(dT) primer and reverse transcriptase (Vazyme Biotech, R312-01). Quantitative reactions were conducted on a CFX96 Touch Real-Time PCR Detection System (Bio-Rad), using the ChamQ SYBR qPCR Master Mix (Vazyme Biotech, Q712-02). At least three independent biological replicates were performed for each experimental condition, with each replicate analyzed in technical triplicate. For assessing the accumulation levels of MR and RSMV genomic RNA in *N. benthamiana* and rice, one-step RT-qPCR reactions were performed by amplifying fragments that spanned two genes to detect replication levels, using the HiScript II One-Step qRT-PCR SYBR Green Kit (Vazyme Biotech, P612-01). The expression levels of *OsEF1a* (for rice) or *NbPP2A* (for *N. benthamiana*) served as internal controls, and relative RNA accumulation levels were calculated using the 2^-ΔΔC(t)^ method (*68*). Primers used for RT-qPCR are listed in table S1.

### Transmission electron microscope observation

RSMV infected leaves from rice plants were fixed, dehydrated, and embedded, and thin sections were cut as described previously (*46*). Ultrathin sections (~80 nm) were prepared from the embedded tissues using a Leica UC7 Ultramicrotome and were collected on 3-mm copper slot grids. These sections were then stained with uranyl acetate and lead citrate solution. The stained sections were examined and imaged using a Thermo Fisher Scientific Talos L120C transmission electron microscope operating at an accelerating voltage of 120 kV.

For immunoelectron microscopy, rice leaves were prefixed with 4% paraformaldehyde (in 100 mM phosphate buffer, pH 7.0) and 0.1% glutaraldehyde for 2 hours, followed by postfixation with 2% paraformaldehyde overnight at 4°C. After treatment with 0.1 M glycine for 1 hour, the fixed leaves were dehydrated in graded ethanol at 4°C and embedded in LR White (GE Healthcare, 14381). The embedded leaves were polymerized at −20°C using ultraviolet light. Sections measuring 100 nm were cut and mounted on slot nickel grids using a Leica UC7 Ultramicrotome. The sections were subsequently mounted on formal-supported nickel single slot grids. Initially, the sections were incubated in 100 mM phosphate buffer for 15 min, followed by incubation in a blocking buffer [1% bovine serum albumin (Sigma-Aldrich, V900933) in 100 mM phosphate buffer, pH 7.0] for another 15 min. For single immunosorbent electron microscopy observation of RSMV P protein, sections were incubated with a rabbit antibody specific to RSMV P (1:100, v:v) for 1 hour at room temperature, followed by incubation with a 12-nm gold-conjugated goat anti-rabbit immunoglobulin G (IgG) secondary antibody (Jackson, 111-205-144) solution (1:100, v:v) for an additional hour. In the case of double immunosorbent electron microscopy observation for RSMV P and PI4P, sections were incubated with a mouse antibody against PI4P (Echelon Biosciences, Z-P004; 1 mg/ml) and a rabbit antibody specific to RSMV P (1:100, v:v). Primary and secondary antibodies were visualized using 10-nm colloidal gold goat anti-mouse IgG (Sigma-Aldrich, G7777) and 18-nm colloidal gold donkey anti-rabbit IgG (Jackson, 711-215-152), respectively. After labeling, sections were stained with uranyl acetate and lead citrate prior to examination under the Talos L120C electron microscope. To count the numbers of P and PI4P labeling gold particles, we set a threshold for each image based on signal strength. Images were then converted to binary format and masked, and the number of particles was counted using Fiji/ImageJ.

### Recombinant protein expression and purification

Recombinant proteins were expressed in *Escherichia coli* Rosetta (DE3) cells by cultivating transformed cells in LB medium at 37°C until reaching an OD_600_ (optical density at 600 nm) of 0.6. Protein expression was subsequently induced by adding 0.5 mM isopropyl-β-d-1-thiogalactopyranoside and incubating overnight at 16°C. Protein purification was then conducted at 4°C.

For the purification of GFP, GFP-P, BFP-P, OsARF1-GFP, OsARF1C-mCherry, mCherry-N, and GFP-hFAPP1-PH, the corresponding bacterial cells were collected and resuspended in a buffer containing 30 mM tris-HCl (pH 6.8), 500 mM NaCl, 10% glycerol, and 20 mM imidazole. The cells were lysed by sonication, and after centrifugation, the supernatants were affinity-extracted using HisPur Ni-NTA Resin (Thermo Fisher Scientific, 88221) and subsequently dialyzed into a protein storage buffer consisting of 30 mM tris-HCl (pH 6.8) and 500 mM NaCl. The proteins were then further purified by size exclusion chromatography with a Superdex 200 column (GE Healthcare, USA) in the protein storage buffer. The peak fractions containing the proteins were collected and concentrated using a 30-kDa molecular weight cutoff (MWCO) Centricon (Millipore, UFC9030-08). The final purified protein concentration was determined using the Bradford assay and SDS–polyacrylamide gel electrophoresis (PAGE). The purified proteins were then snap-frozen in small aliquots using liquid nitrogen and stored at −80°C.

To purify glutathione *S*-transferase (GST)–P and maltose binding protein (MBP)-OsARF1C proteins, bacterial cells were collected, resuspended in 1× phosphate-buffered saline (PBS) buffer (pH 7.0), and lysed by sonication. Following centrifugation, the supernatants were affinity-extracted using Glutathione Sepharose 4B (GE Healthcare, 17075601) and Amylose Resin (New England Biolabs, E8021V), respectively. Subsequently, the purified proteins were concentrated using a 30-kDa MWCO Centricon (Millipore, UFC903008).

### In vitro liquid droplet reconstitution assay

For the phase separation of GFP-P or BFP-P, the proteins were diluted to the desired concentrations using 30 mM tris-HCl (pH 6.8), with a final NaCl concentration of 50 mM unless otherwise specified. For the phase separation of mCherry-N/OsARF1C-mCherry in combination with GFP-P, the proteins were mixed and then diluted to the desired concentrations in 30 mM tris-HCl (pH 6.8). In the case of PI4P with GFP-P, GFP-P was diluted in a buffer containing PI4P [30 mM tris-HCl (pH 6.8) and 100 μM PI4P/PI (Echelon Biosciences, P-4016/P-0016)]. Similarly, for the phase separation of PI4P with GFP-hFAPP1-PH and BFP-P, the two proteins were mixed and diluted in a buffer containing PI4P [30 mM tris-HCl (pH 6.8) and 100 μM PI4P/PI (Echelon Biosciences, P-4016/P-0016)]. Thoroughly mixed reactions were transferred into a 384-well plate, and after 1 hour of incubation at room temperature, the resulting droplets in the wells were observed using confocal microscopy (Leica TCS SP8). For the synthesis of Cy5-labeled RNAs, 5 to 10 μg of DNA templates containing the T7 promoter–driven RSMV trailer sequence were used as templates for in vitro transcription, using the T7 High Yield RNA Synthesis Kit (Yeasen, 10623ES10) according to the manufacturer’s protocols. The final concentrations of adenosine triphosphate, cytidine triphosphate, GTP, uridine triphosphate (UTP), and Cy5-UTP (Yeasen, 10623ES10) in the reaction mixtures were 1.75, 1.75, 1.75, 0.875, and 0.175 mM, respectively. The reactants were mixed gently and incubated at 37°C for 2 hours, followed by the addition of RNase-Free DNase I for 15 min to eliminate DNA templates. Subsequently, 0.1 volumes of 3 M sodium acetate (pH 5.2) and 2.5 volumes of 100% ethanol were added, and the mixture was stored at −20°C for over 4 hours. After centrifugation, the precipitated RNA was washed with 75% ethanol, resuspended in nuclease-free water, and heated at 95°C for 5 min. The phase separation of GFP-P, mCherry-N, and Cy5-labeled RNAs was performed by mixing the indicated proteins with Cy5-labeled RNAs, followed by dilution with buffer [30 mM tris-HCl (pH 7.5) and 1 mM dithiothreitol (DTT)] to achieve the desired concentrations.

BFP, GFP, and mCherry fluorescence were excited/visualized under 405 nm/415 to 488 nm, 488 nm/500 to 530 nm, and 543 nm/588 to 641 nm, respectively. To quantify the number and size of GFP-P droplets, we established a threshold for each image based on signal strength. Subsequently, the images were converted to binary format and masked, and the number and size of droplets were quantified using Fiji/ImageJ.

### FRAP assay

FRAP of GFP-P granules in *N. benthamiana* leaf epidermal cells was conducted using a Leica TCS SP8 confocal microscope equipped with a 40× objective lens. The GFP-P granules were subjected to photobleaching with a laser intensity of 100% at 488 nm over 50 iterations. Fluorescence recovery was monitored every 3 s for a duration of 120 s following the bleaching process. Ten droplets were used to obtain the FRAP recovery curves.

FRAP of GFP-P droplets in a 384-well plate was conducted using a Leica TCS SP8 confocal microscope equipped with a 100× oil-immersion objective. The central region of each GFP-P droplet was subjected to bleaching using a laser intensity of 100% at 488 nm, with a total of 100 iterations. Fluorescence recovery was monitored every 50 s for a duration of 550 s following the bleaching process. Ten droplets were used to obtain the FRAP recovery curves.

### Y2H assay

For the Y2H assay, different combinations of pGBK and pGAD plasmids were transformed into the yeast strain Y2HGold. The transformants were initially cultivated on synthetic defined (SD)–Trp/−Leu plates for 2 days. Subsequently, the transformants were cultivated on SD/-Leu/-Trp (SD-L-T) medium and lastly cultivated on SD/-Leu/-Trp-His-Ade (SD-L-T-H-A) selection medium to determine the protein-protein interaction. Yeast cells were photographed 3 days postincubation at 30°C.

### GST pull-down assay

A total of 3 μg of purified GST-P protein was mixed with 6 μg of purified MBP-OsARF1C or MBP protein and incubated with 20 μl of Glutathione Sepharose 4B beads (GE Healthcare, 17075601) for 2 hours at 4°C in binding buffer [50 mM tris-HCl (pH 7.5), 150 mM NaCl, and 0.25% Triton X-100]. After washing four times with binding buffer, the resin-bound proteins were released by boiling for 5 min, and the protein samples were analyzed using an immunoblot assay with anti-GST and anti-MBP antibodies.

### Co-IP assay

Immunoprecipitation (IP) was conducted using protein extracts derived from *OsARF1C-OE* or WT plants with RSMV or mock infection. At 15 dpi, the rice leaves were ground in liquid nitrogen, and protein samples were extracted using an IP buffer consisting of 40 mM tris-HCl (pH 6.8), 150 mM NaCl, 5 mM MgCl_2_, 2 mM EDTA, 5 mM DTT, 2% glycerol, 0.1% Triton X-100, and protease inhibitor cocktail from 5 g of leaves. Following a 10-min incubation at 4°C, the mixtures were centrifuged at 12,000*g* for 10 min. The supernatant was then mixed with 1 μl of anti-P antibody and incubated for 2 hours at 4°C, followed by the addition of 20 μl of Protein A+G Agarose (Beyotime Technology, P2055). The immunoprecipitants were washed four times with 1× PBS and resuspended in 20 μl of 2× SDS-PAGE loading buffer, which consisted of 500 mM tris-HCl (pH 6.8), 50% glycerol, 10% SDS, 2% β-mercaptoethanol, and 1% bromophenol blue. The protein samples were then boiled at 95°C for 10 min and separated using a 10% SDS-PAGE gel for subsequent Western blot analysis.

### Subcellular localization and BiFC assay

*N. benthamiana* and rice protoplasts were used in BiFC and subcellular localization assays. Various combinations of expression vectors were transiently expressed in *N. benthamiana* leaves via agroinfiltration for 55 to 60 hours or transfected into rice protoplasts for 12 to 16 hours. YFP fluorescence indicated in vivo protein interactions. RFP-H2B, expressed in transgenic *N. benthamiana* leaves, served as a nuclear marker. Cyan fluorescent protein/BFP, GFP, YFP, and RFP/mCherry fluorescence were excited/visualized under 405 nm/415 to 488 nm, 488 nm/500 to 530 nm, 514 nm/525 to 580 nm, and 543 nm/588 to 641 nm, respectively.

### Chemical treatment

To inhibit GFP-P droplets in vivo and vitro, we treated 10% of HEX for 5 min in GFP-P–expressed *N. benthamiana* leaves and added 5% of HEX to GFP-P–contained elution buffer for 1 min before observation.

*N. benthamiana* leaves were infiltrated with the RSMV MR system to inhibit ARF1 and PI4KB activity. At 5 dpi, the leaves received treatments with dimethyl sulfoxide (DMSO), BFA (Aladdin, B102375; final concentration of 50 μg/ml), or PIK93 (Aladdin, P126888; final concentration of 100 μM). Following a 24-hour incubation, the cells were examined using confocal microscopy.

### P protein–PI4P complex modeling

The Chai-1 modeling framework (www.chaidiscovery.com) was used for predicting protein-ligand complex structures. Protein sequences in FASTA format and ligands in SMILES format were inputted into Chai-1, which combines protein folding prediction with ligand docking simulation. Five candidate structures were generated for each complex and ranked based on structural feasibility and interaction quality. Key binding interactions such as hydrogen bonds, hydrophobic contacts, and potential covalent binding sites were identified. The final model was visualized using ChimeraX 1.9, highlighting binding sites and key interactions to elucidate the ligand’s binding mode effectively.

### Protein-lipid binding assay

PI4P or PI was dissolved in a buffer consisting of chloroform, methanol, and water in a 1:2:0.8 ratio, achieving a concentration of 1 mM. This solution was then spotted onto a nitrocellulose membrane (Whatman Protran, BA83) at a predetermined concentration. Subsequently, the nitrocellulose membranes were blocked with 1% fatty acid–free milk in 1× tris-buffered saline (20 mM tris and 150 mM NaCl, pH 8.0) for 1 hour. Following the blocking step, the membranes were incubated with purified His-tagged proteins (GFP-P, ARF1C-GFP, GFP-hFAPP1-PH, and GFP) at a concentration of 2 μg/μl for 3 hours at room temperature. After washing, the membranes were incubated with mouse monoclonal anti–6× His tag (Abmart) for 2 hours, followed by incubation with goat anti-mouse IgG–horseradish peroxidase antibody (Mei5bio) for 1 hour at room temperature. Last, the strips were analyzed using a chemiluminescence gel imaging system (Bio-Rad, USA).

### Western blot and protein quantification analysis

Individual protein samples were extracted using radioimmunoprecipitation assay lysis buffer. The samples were centrifuged at 13,400*g* for 2 min, and the upper liquid phase of each sample was analyzed by SDS-PAGE. The PageRuler Prestained Protein Ladder (Thermo Fisher Scientific, 26616) was used to determine the protein sizes. The separated protein bands were transferred to polyvinylidene difluoride membranes (Millipore, Billerica, MA) and detected using antibodies against RSMV N (from J. Wu), RSMV P (generated in this study, produced by Abmart), His (Abmart, M30111), GST (Abmart, M20007), MBP (Abmart, M20051), Myc (Abmart, M20002), GFP (Abmart, M20004), RFP (ABclonel, WH133973), tubulin (Abmart, M20045), or actin (Abmart, M20009). For protein band quantification, the signal intensity was quantified using Fiji/ImageJ. The actin or tubulin bands served as the loading control, and the intensity of individual protein bands was normalized against that of the actin band from the same sample.

### Statistics and reproducibility

All experiments were independently repeated more than three times, yielding consistent results. No data were excluded from our studies. Differences were analyzed using either one-way or two-way analysis of variance (ANOVA), followed by Tukey’s multiple comparisons test. A *P* value of less than 0.05 was considered significant. All analyses were conducted using SPSS version 20 (SPSS, Inc., Chicago, IL, USA).
