# Supplementary material for: Plant negative-strand RNA virus phosphoprotein condensates exploit host trafficking and lipid synthesis for viral factory assembly
Source: Sci Adv. 2025 Aug 20;11(34):eadx7905. doi: 10.1126/sciadv.adx7905 (PMC12366689; doi:10.1126/sciadv.adx7905)
Supplement: Supplementary file 1 — Figs. S1 to S7 Table S1 Legend for movie S1 [file sciadv.adx7905_sm.pdf]

Science Advances
AAAS

Supplementary Materials for

# Plant negative-strand RNA virus phosphoprotein condensates exploit host trafficking and lipid synthesis for viral factory assembly

Zhiyi Wang *et al.*

Corresponding author: Tong Zhang, zhangtong@scau.edu.cn; Guohui Zhou, ghzhou@scau.edu.cn

*Sci. Adv.* **11**, eadx7905 (2025)
DOI: 10.1126/sciadv.adx7905

**The PDF file includes:**

Figs. S1 to S7
Table S1
Legend for movie S1

**Other Supplementary Material for this manuscript includes the following:**

Movie S1

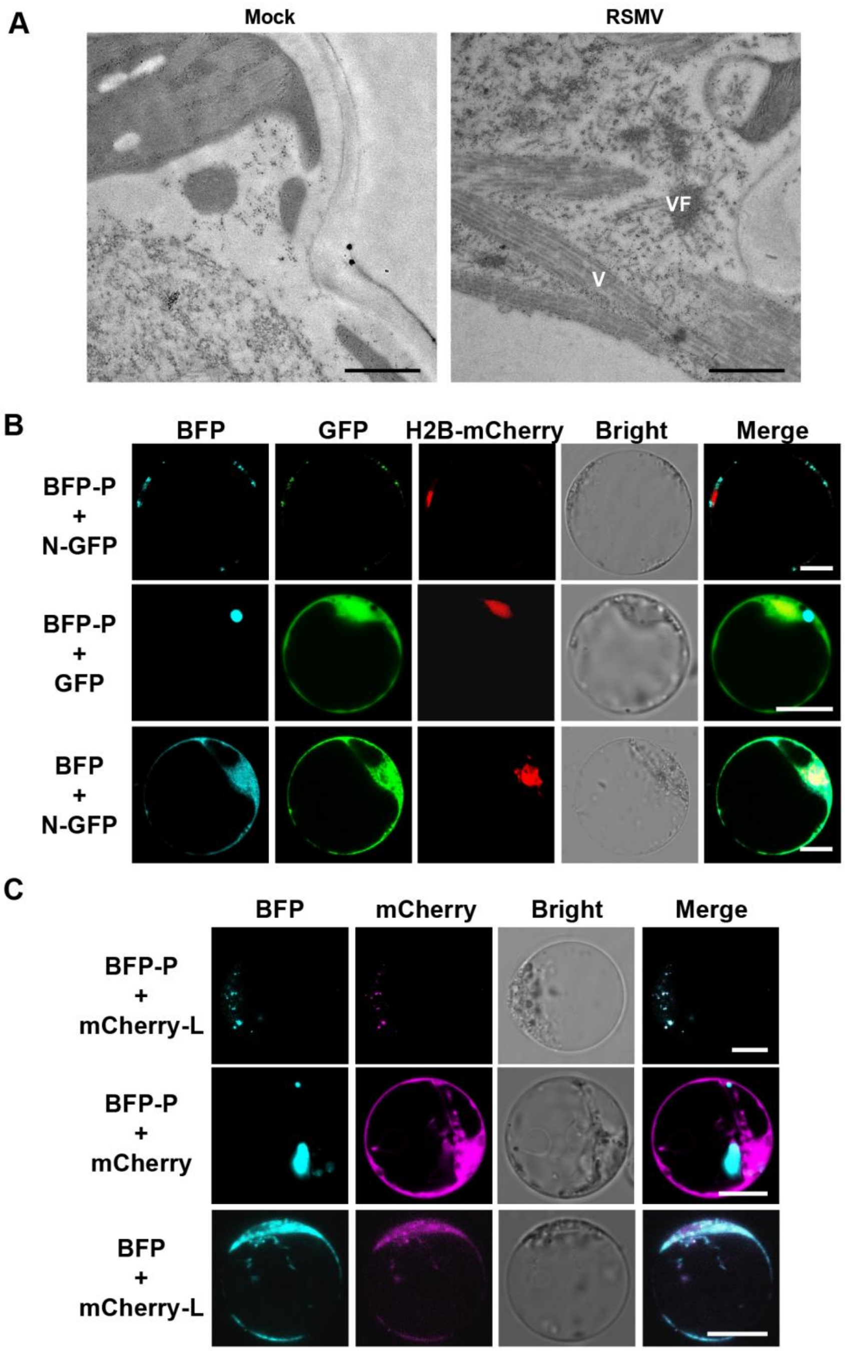


**Fig. S1. RSMV P is a key component of VFs (related to Fig. 1).**

**A** Transmission electron microscopy characterization of the RSMV viroplasm in rice leaves infected by RSMV at 30 days post infiltration (dpi). V indicates virions; VF indicates viral factory. Healthy stems served as a negative control. Scale bar = 200 nm. **B** Subcellular distribution of BFP-P and N-GFP in rice protoplasts. Scale bar = 10 μm. **C** Subcellular distribution of BFP-P and mCherry-L in rice protoplasts. Scale bar = 10 μm.

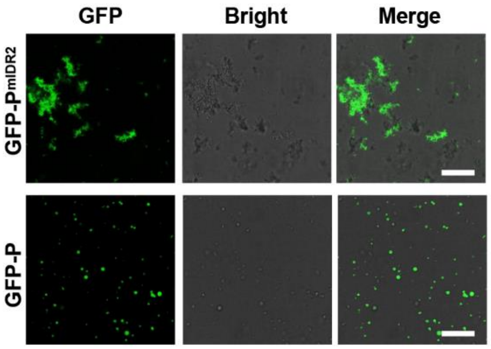


**Fig. S2. The IDR2 of P is involved in the LLPS of the protein (related to Fig. 2).**
Confocal images showing droplets formed by GFP-$P^{mIDR2}$ and GFP-P in vitro. Scale bar = 20 μm.

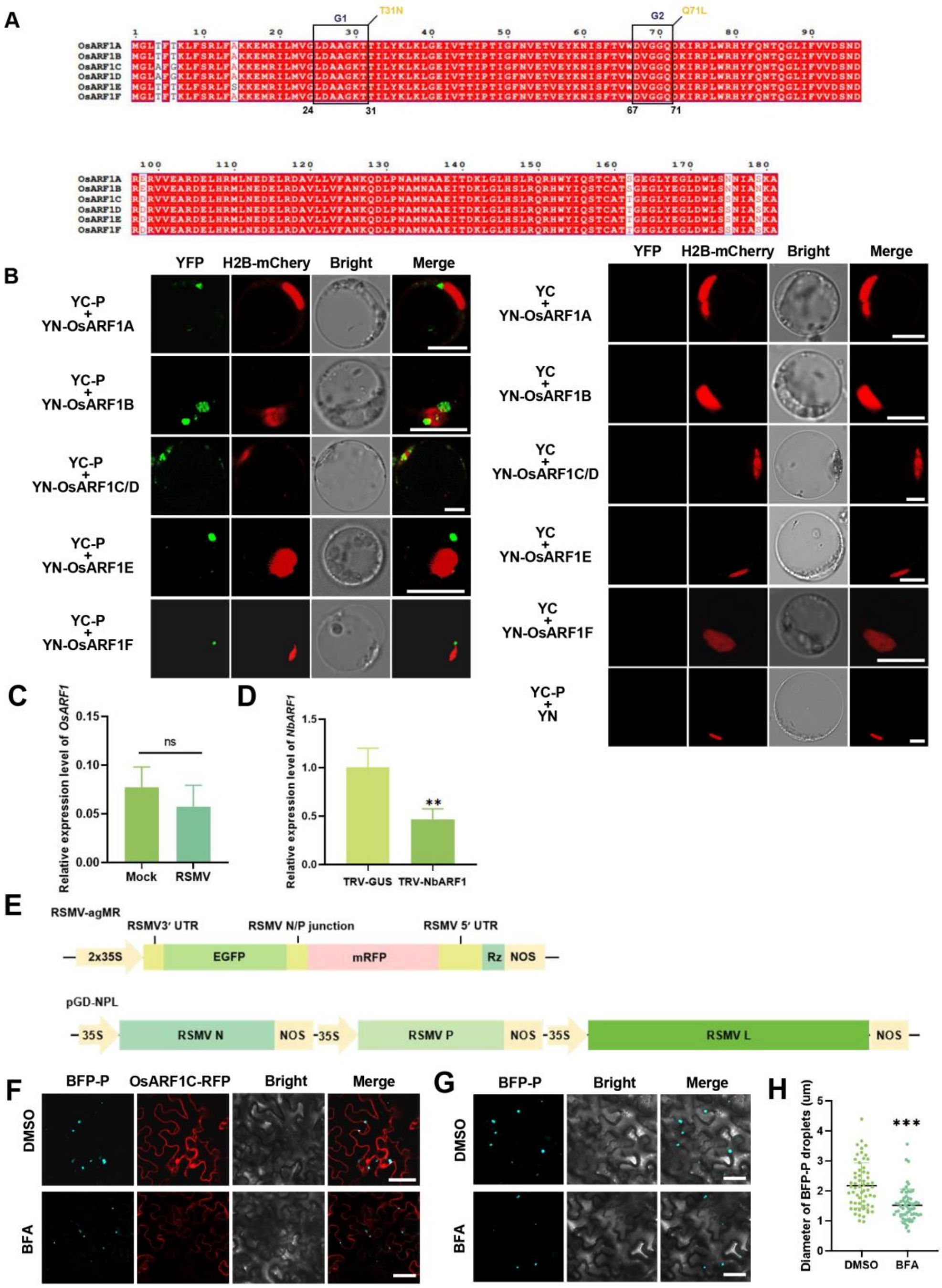


**Fig. S3. RSMV P interacts with all six homologs of OsARF1 (related to Fig. 3).**

**A** Alignment of ARF1 protein sequences of rice using the ESPript 3.0 website; red areas indicate identical amino acids between proteins, while white areas represent differing amino acids. **B** BiFC assay confirming that protein P interacts with six OsARF1 variants in rice protoplasts. Scale bar = 10 µm. **C** RT-qPCR analysis of OsARF1 gene expression levels in RSMV-infected and healthy rice plants. The values shown are the means ± SD (n = 4). A Student's *t*-test was applied for analysis; ns indicates no significant difference between the two groups. **D** Silencing efficiency of NbARF1 evaluated by qRT-PCR. Student's *t*-test was applied for analysis. The values shown are

the means ±SD (n = 4). ** indicates a highly significant difference between the two groups ($p < 0.01$). **E** Schematic representation of the plasmids designed to generate antigenomic-sense RSMV derivatives and to express the RSMV N, P, L, and VSR proteins in vivo. In the agMR plasmid, a reporter cassette of antigenomic-sense or genomic-sense RSMV derivative was inserted between the CaMV double 35S promoter (2X35S) and the ribozyme sequence (RZ). UTR, untranslated region; RSMV NP junction, intergenic sequences including N 3' UTR, intergenic sequence, and P 5' UTR; Nos, nopaline synthase terminator. **F** Effects of BFA treatment for subcellular localization of BFP-P and OsARF1C-RFP in *N. benthamiana* leaves. **G** Confocal images depicting BFP-P granules under BFA/DMSO treatment in *N. benthamiana* leaves. Scale bar = 10 μm. **H** Statistical analysis of BFP-P granule diameter in panel (G), with at least 50 BFP-P granules measured. A Student's t-test was utilized for the analysis, with *** denoting a highly significant difference between the two groups of data ($p < 0.001$).

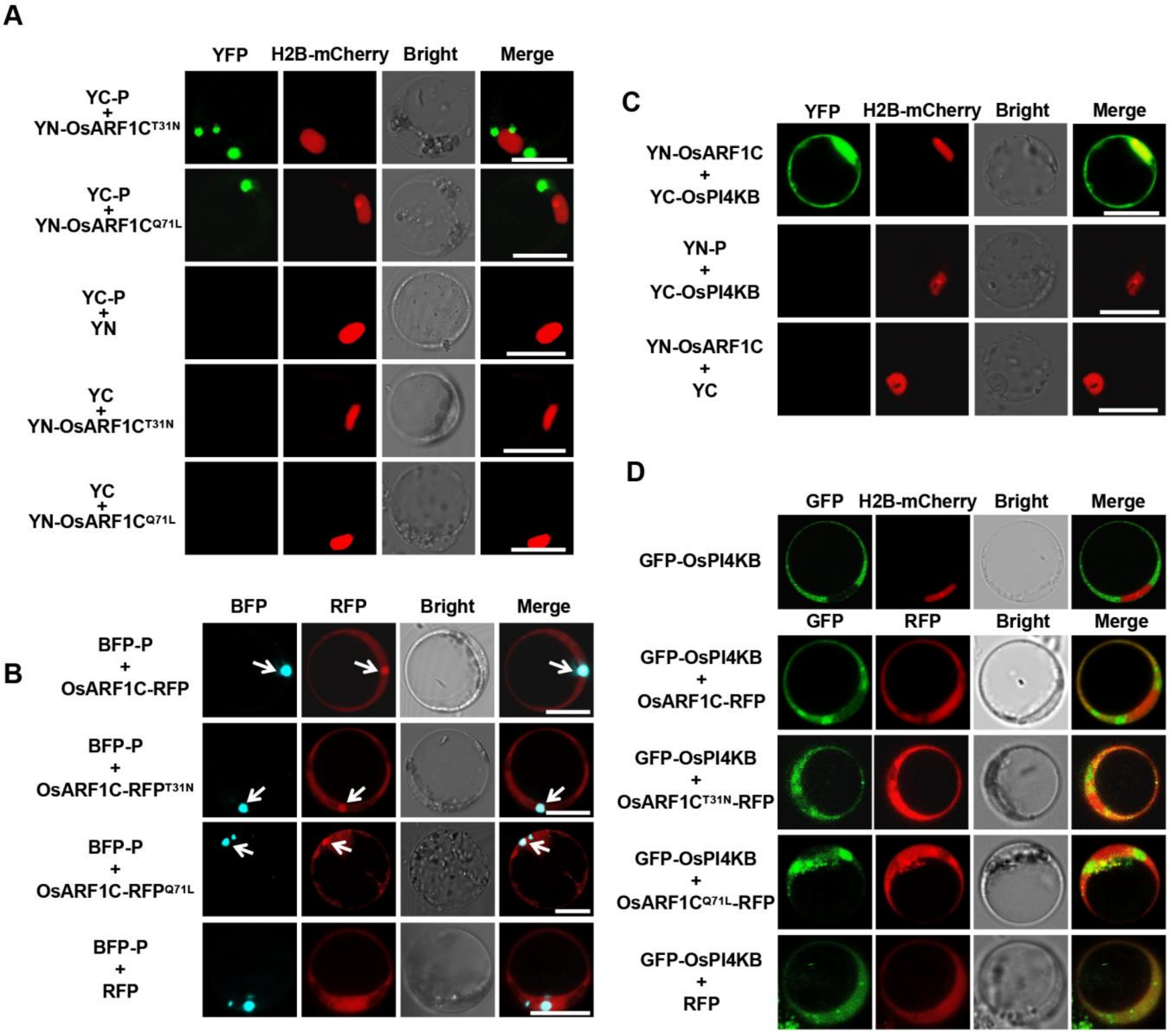


**Fig. S4. Interaction between P, OsARF1C, and OsPI4KB (related to Fig. 5).**

**A** BiFC assay confirms that P interacts with OsARF1C$^{T31N}$ and OsARF1C$^{Q71L}$ in rice protoplasts, respectively. Scale bar = 10 μm. **B** Confocal images displaying the subcellular localization of P and point mutants of OsARF1C (OsARF1C$^{T31N}$ and OsARF1C$^{Q71L}$) in rice protoplasts, respectively. Scale bar = 10 μm. **C** BiFC assay confirming that OsARF1C interacts with OsPI4KB in rice protoplasts. Scale bar = 10 μm. **D** Confocal images depicting the subcellular localization of OsPI4KB and the point mutants of OsARF1C (OsARF1C$^{T31N}$ and OsARF1C$^{Q71L}$) in rice protoplasts, respectively. Scale bar = 10 μm.

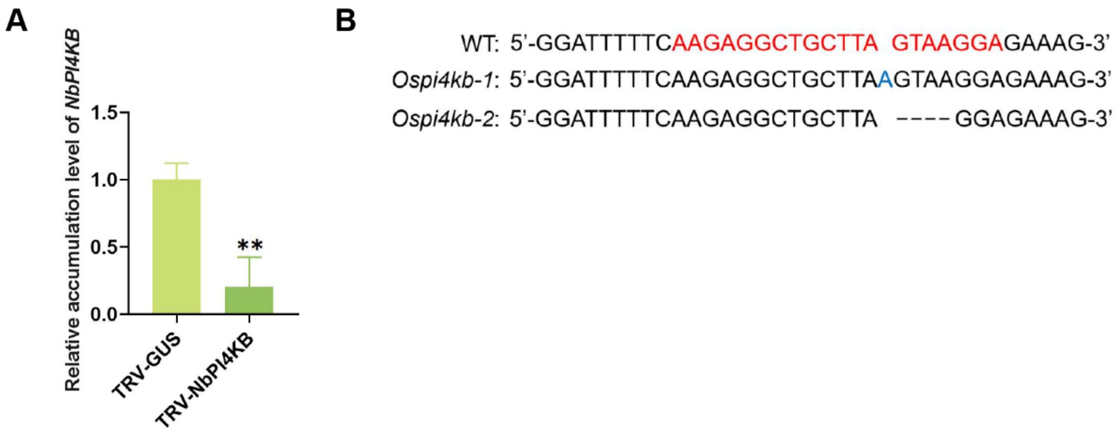


**Fig. S5. Silencing *NbPI4KB* and Generation of *OsPI4KB*-mutant rice plants (related to Fig. 6).**

**A** Silencing efficiency of NbPI4KB assessed by qRT-PCR. The values shown are the means ± SD (n = 4). Student's *t*-test was employed for analysis, where ** indicates a highly significant difference between the two groups of data ($p < 0.01$). **B** CRISPR/Cas9-mediated target mutagenesis of OsPI4KB. This panel shows the alignment of the WT, *Ospi4kb-1*, and *Ospi4kb-2* sequences containing the CRISPR/Cas9 target site. A 20-bp CRISPR/Cas9 target sequence adjacent to the underlined PAM is indicated in red in the WT sequence. The newly created Ospi4kb-1 and Ospi4kb-2 mutants exhibit a 1-bp insertion (A, in blue font) and a 4-bp deletion (GTAA, shown as a dash), respectively.

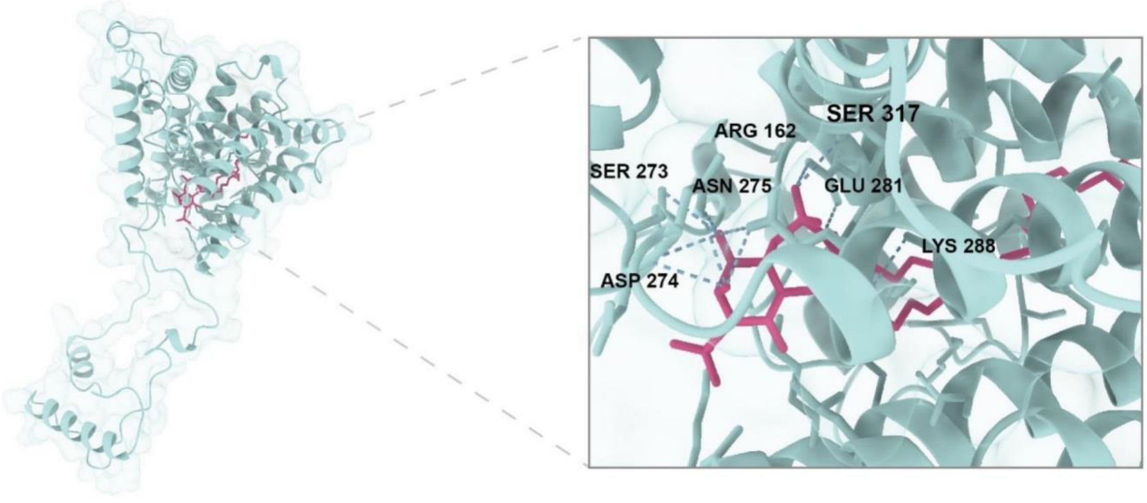


**Fig. S6. The molecular docking model of RSMV P and PI4P (related to Fig. 7).**

Chai-1 was employed to demonstrate the molecular docking model of RSMV P (green) and PI4P (red).

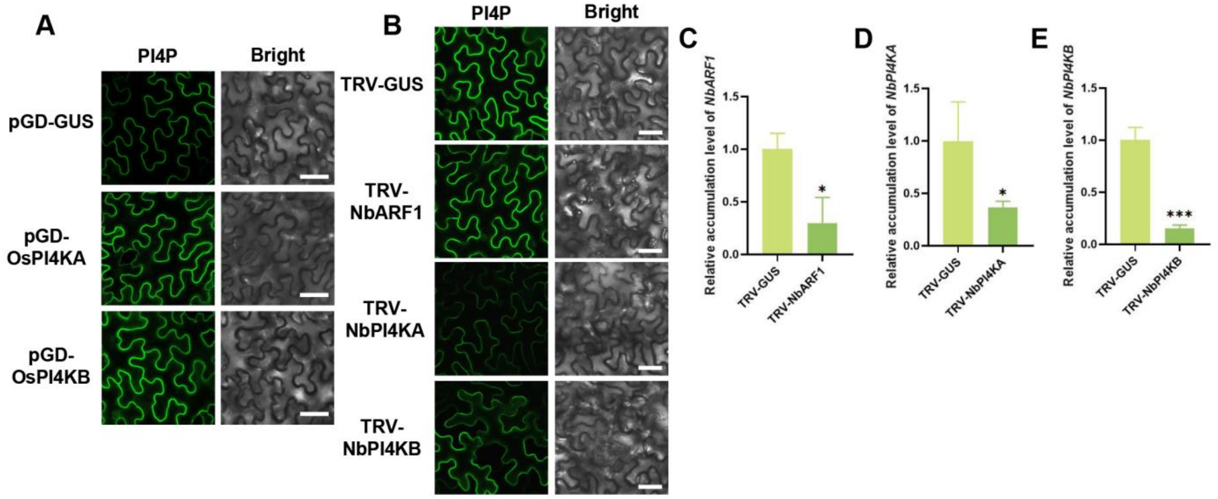


**Fig. S7. The levels of PI4P were monitored in *N. benthamiana* leaves with silenced or expressed PI4K (related to Fig. 8).**

**A** Confocal image showing PI4P fluorescence intensity in *N. benthamiana* plants inoculated with TRV-GUS, TRV-NbARF1, TRV-NbPI4KA, and TRV-NbPI4KB, respectively. Scale bar = 10 μm. **B-D** Silencing efficiency of NbARF1, NbPI4KA, and NbPI4KB, measured by qRT-PCR. Student's *t*-test was employed for statistical analysis. The values shown are the means ± SD (n = 4). * and ** indicate significant differences between the two groups of data, with $p < 0.05$ and $p < 0.01$, respectively.

**Table S1.**
**Primers used in this study.**

| Primer Name | Primer Sequence (5’-3’) |
|---|---|
| qMR-F | GGGATCACTCTCGGCATGGAC |
| qMR-R | GCACCTTGAAGCGCATGAAC |
| PP2A-F | GACCCTGATGTTGATGTTCGCT |
| PP2A-R | GAGGGATTTGAAGAGAGATTTC |
| pGADT7-OsARF1-F | GCCATGGAGGCCAGTGAATTCATGGGGTTGGCGTTCGGGA |
| pGADT7-OsARF1-R | ACGATTCATCTGCAGCTCGAGTCAAGCCTTGCTAGCAATGT TGCTG |
| pGBKT7-P-F | ATGGCCATGGAGGCCGAATTCATGAGTGTGCCAGAGGATA CTCCC |
| pGBKT7-P-R | CCGCTGCAGGTCGACGGATCCCCAGTGCATCATCATAGTAA TCCGATG |
| pENTR-P-F | CCGCGGCCGCCCCCTTCACCATGAGTGTGCCAGAGGATACT C |
| pENTR-P-nsR | GGGTCGGCGCGCCCACCCTTCAGTGCATCATCATAGTAATC C |
| pGEXT-P-F | CCGCGTGGATCCCCGGAATTCATGAGTGTGCCAGAGGATA CTCCC |
| pGEXT-P-R | GTCACGATGCGGCCGCTCGAGTCACAGTGCATCATCATAGT AATCCG |
| pMBP-OsARF1c-F | TATCGGAATTAATTCGGATCCGATGGGGTTGGCGTTCGGGA |
| pMBP-OsARF1c-R | GTGGTGGTGGTGGTGCTCGAGTCAAGCCTTGCTAGCAATGT TGCTG |
| pENTR-OsARF1c-F | CCGCGGCCGCCCCCTTCACCATGGGGTTGGCGTTCGGGA |
| pENTR-OsARF1c-R | GGGTCGGCGCGCCCACCCTTAGCCTTGCTAGCAATGTTGCT G |
| Seq-GST-F | GGGCTGGCAAGCCACGTTTGGTG |
| Seq-MBP-F | ACTGATGAAGGTCTGGAAGCGG |
| Seq-T7-F | TAATACGACTCACTATAGGGC |
| T31N-F | CGGAAAGAACACCATCCTC |
| T31N-R | GAGGATGGTGTTCTTTCCG |
| Q71L-F | TCGGGGGTCTGGACAAGAT |
| Q71L-R | ATCTTGTCCAGACCCCCGA |

| | |
|---|---|
| pRHV-myc-OsARF1C-F | ATCCCCGGGTGAGCTCATGGGGTTGGCGTTCGGGA |
| pRHV-myc-OsARF1C-R | CGCACTAGTAAGCTTAGCCTTGCTAGCAATGTTGCT |
| qPRHV-OsARF1C-F | GGTACATCCAGAGCACTTGTGCC |
| HRG-ubi-RNAi ARF1-F1 | ACTTCTGCAGAAGCTTCTGATGGTCGGTCTCGAC |
| HRG-ubi-RNAi ARF1-R1 | GCGGTATATACGTACGGTACCAGCTTCCACAACACGGTC |
| HRG-ubi-RNAi ARF1-F2 | GTACGTGTGCAGGGATCCAGCTTCCACAACACGGTC |
| HRG-ubi-RNAi ARF1-R2 | CGAGCTGGTCAGAGCTCCTGATGGTCGGTCTCGAC |
| RNAi-QCR | AAAAATTAGGCATCATCAG |
| RNAi-QCF | TATATGTATGCGTCCAGTCCA |
| ubi-seqF | CAGCAGCTATATGTGGATTT |
| qOsARF1-R | CGACAACACGGTCCCTATCA |
| TRV-NbARF1-F | GAAGGCCTCCATGGGGATCCCTGTGCTGCTTGTTTTTGCT |
| TRV-NbARF1-R | GGACATGCCCGGGCCTCGAGCTTCGTTTACAAATTTATG |
| qNbARF1-F | AATGACAGAGACCGTGTTGTTGA |
| qNbARF1-R | ACAGCATCCCGAAGCTCATC |
| TRV-NbPI4KA-F | GAAGGCCTCCATGGGGATCCATTGTTCACAGGGCTTTTGAAA |
| TRV-NbPI4KA-R | GGACATGCCCGGGCCTCGAGAGTACTGAGCACCTCTAAATTAAAC |
| qNbPI4KB-F | ACCAGTCACTTGATTTTGTGCAG |
| qNbPI4KB-R | TGTGCATTGATCTCCGCTAGAG |
| qNbPI4KA-F | CCAACGCATTTCTCAAGGAAC |
| qNbPI4KA-R | TTCTCATTGCCACTACCACG |

| | |
|---|---|
| TRV-NbPI4KB-F | GAAGGCCTCCATGGGGATCCCCATCTCTTCCATTGAAGTCAG |
| TRV-NbPI4KB-R | GGACATGCCCGGGCCTCGAGTTCCCAGAGTTCTCCAGAAAG |
| pGD-EGFP-OsPI4KB-F | TCTATCTCTGGATCCATGGTGAGCAAGGGCGAGGA |
| EGFP-OsPI4KB-R | GAGAAGCCGCACCATACCGCTGCTACCCTTGTACAGCTCGTCCATG |
| pGD-OsPI4KB-R | ACGAGCTCTGTCGACTCATAAAATCCCATTCAATACTCTCTGGTAGTAA |
| GSSG-OsPI4KB-F | GGTAGCAGCGGTATGGTGCGGCTTCTCGGG |
| pGD-YC-OsPI4KB-F | TCTATCTCTGGATCCGGCAGCGTGCAGCTC |
| YC-OsPI4KB-R | GAGAAGCCGCACCATACCGCTGCTACCCTTGTACAGCTCGTCCATGC |
| EX108-hFAPP1-PH-F | TCAGCAGTCGAAGAGCATGGAGGGGGTGTTGTACAAG |
| EX108-hFAPP1-PH-R | TTAGCGTGTGAAGAGCTGTATCAGTCAAACATGCTTTGGAGC |
| pET28a-mGFP-FAPPH/P-F | CGCGGATCCGAATTCATGGTGAGCAAGGGCGAGGA |
| pET28a-mGFP-FAPPH-R | TTGTCGACGGAGCTCGATGTATCAGTCAAACATGCTTTGGAGC |
| pET28a-BFP-P-F | CGCGGATCCGAATTCATGGTGTCTAAGGGCGAAG |
| pET28a-BFP/mGFP-P-R | TTGTCGACGGAGCTCGATCACAGTGCATCATCATAGTAATCCG |
| P6end-F | GGATCCTCACAATGGGTACTGC |
| RSMV b/R | GTCACCAGAGCAGACCTCAG |
| T7-RSMV Trailer R | TAATACGACTCACTATAGGGAAGGAAGTTGTGTGTTGCG |
| RSMV Trailer F | ATGGATGATTTAATGGTCTATAGCCTT |
| mGFP-P-R | ACTCATTCCACTACTTCCTTTGTATAGTTCATCCATGCCAT |
| mGFP-P-F | ATACAAAGGAAGTAGTGGAATGAGTGTGCCAGAGGATAC |

| | |
|---|---|
| BFP-P-R | GCCGCCGCCGCCGCTGCCGCCGCCGCCATTAAGCTTGTGCCCCAGTT |
| BFP-P-F | GCTGCCGCCGCCGCCGCTGCCGCCGCCGCCATTAAGCTTGTGCCCCAGTT |
| pET28a-mCherry-F | ATGGGTCGCGGATCCGAATTCATGGTGAGCAAGGGCGAGGA |
| mCherry-ARF1-R | AACCCCATTCCACTACTTCCAGATCTGTACAGCTCCTCCAT |
| mCherry-ARF1-F | ACAGATCTGGAAGTAGTGGAATGGGGTTGGCGTTCGGGAA |
| pET28a-ARF1-R | GCAAGCTTGTCGACGGAGCTCTCAAGCCTTGCTAGCAATGT |
| pET28a- mCherry-F | ATGGGTCGCGGATCCGAATTCATGGTGAGCAAGGGCGAGGA |
| N- mCherry-R | TGCCATTCCACTACTTCCAGATCTGTACAGCTCCTCCA |
| mCherry-N-F | AGATCTGGAAGTAGTGGAATGGCAACCGACAAGTCTTT |
| pET28a-N-R | GCAAGCTTGTCGACGGAGCTCTTAAGCCTTGGTCTGGAAGAT |

**Movie S1.**

High mobility of RSMV GFP-P inclusion bodies in *N. benthamiana* leaves.
